# Dynamic Porosity Characteristics of Corn Bed During Convective Drying: A Numerical and Experimental Study Based on EDEM–Fluent Coupled Simulation

**DOI:** 10.3390/foods15203615

**Published:** 2026-10-11

**Authors:** Xuefeng Zhang, Qiao Li, Hao Wu, Xiaoyong Yang, Zebao Li, Yuelang Liu, Bin Li

**Affiliations:** College of Intelligent and Manufacturing Engineering, Chongqing University of Arts and Sciences, Chongqing 402160, China; 20230015@cqwu.edu.cn (X.Z.); 18523671019@163.com (Q.L.); 202501015037@stu.cqwu.edu.cn (H.W.); 17623078947@163.com (X.Y.); 202401015034@cqwu.edu.cn (Z.L.); 202401015016@cqwu.edu.cn (Y.L.)

**Keywords:** corn, dynamic porosity, EDEM–Fluent

## Abstract

During the grain drying process, porosity directly affects the pore curvature, effective evaporation area, and ventilation resistance of the granular bed, which is an important factor in determining the drying rate, energy consumption and drying uniformity. Existing research on the porosity of flowing maize beds is relatively limited and has primarily focused on static pile conditions. Therefore, this study designed an air convection measurement system consisting of a variable-frequency fan, a valve opening adjustment device, and a differential pressure sensor. The following factors were selected: moisture content (13.03–33.37% w.b.), air velocity (0.6–1.0 m/s), maize flow velocity (0.04–0.06 m/s), and bed thickness (20–40 cm). Experiments were conducted using a Box–Behnken design, and the measured results were compared with theoretical values from the Ergun equation and results from EDEM–Fluent coupled simulations. The dynamic porosity ranged from 57.81% to 63.30%. Porosity decreased as moisture content and pile thickness increased, while it increased as the corn flow velocity increased; air speed had a minor effect. The response surface model had an *R*^2^ value of 0.9600, and the relative deviations between the measured, simulated, and predicted values did not exceed ±1%. The conclusion is that water content, corn flow velocity, and pile thickness are the primary factors affecting dynamic porosity.

## 1. Introduction

Corn is one of the major food and economic crops in China. Safe drying and storage are essential for reducing postharvest losses and maintaining grain quality. A bulk corn bed consists of solid kernels and interconnected voids, and can therefore be regarded as a granular porous medium [1,2]. The pore structure determines the available flow space for air and affects airflow resistance, heat transfer, and moisture migration within the bed [2,3,4]. These transport processes are closely related to grain drying and storage [5,6]. Reducing postharvest grain losses is also important for food security [7]. Porosity refers to the ratio of the internal void volume to the total volume of granular agricultural products, such as wheat, rice, and corn. It can directly reflect the compactness of the bed and affect effective airflow and ventilation resistance. Meanwhile, particle sliding, collision, and rearrangement in a dynamic bed continuously change the pore structure. Besides porosity, parameters such as particle size and shape, bulk density, permeability, and moisture content also affect airflow, and heat and mass transfer within the granular bed. Factors such as porosity, particle size and shape, and particle motion are closely related to grain storage [8]. During grain flow, continuous particle movement changes the packing arrangement and may further alter the pore structure of the corn bed.

Considerable research has been conducted on the pore structure and transport characteristics of stationary grain beds. Ge [9] investigated the pore structure and heat and moisture transfer characteristics of maize bulk. Li et al. [10] analyzed local heat and mass transfer in barley grain piles at the pore scale and reported lower transfer rates in regions with lower porosity. Chen et al. [11] studied the ventilation resistance of maize beds and examined the relationship between pressure loss, bed thickness, and porosity. Chen et al. [12] further investigated the effect of vertical pressure on heat and moisture transfer in wheat bulk. CT-based analysis of granular filter media also indicated that particle shape affects pore geometry and structural characteristics [13]. These studies provide a basis for understanding the relationship between pore structure and transport behavior in granular beds. However, most of the reported work was conducted under stationary or fixed-bed conditions. The effect of continuous particle movement and rearrangement on bed porosity has received much less attention.

Numerical methods based on particle-scale modeling have also been applied to grain-bed systems. Liu et al. [14] used the discrete element method (DEM) to construct fixed beds of non-spherical soybean, wheat, and maize particles, and combined the particle models with computational fluid dynamics (CFD) to analyze airflow and heat transfer. Their results showed non-uniform distributions of porosity and airflow within fixed grain beds. Mousaviraad and Tekeste [15] developed DEM models for corn at different moisture contents, and validated the models through hopper-discharge and particle-impact tests. Their work reproduced corn flow behavior under different moisture conditions. However, the former study considered a fixed particle bed, while the latter mainly focused on particle flow during discharge. Neither study directly quantified bed porosity under the combined conditions of continuous corn flow and airflow. Therefore, dynamic porosity under coupled particle movement and airflow remains insufficiently understood. The combined effects of moisture content, corn flow velocity, air velocity, and bed thickness also require further investigation. Experimental validation is also needed to determine whether an EDEM–Fluent coupled model can accurately represent the dynamic porosity of a flowing corn bed.

To address these limitations, this study developed a self-built experimental platform for measuring the dynamic porosity of a flowing corn bed. Corn kernels moved downward through the measurement zone, while air flowed upward through the bed. Four operating factors were considered: moisture content (13.03–33.37% w.b.), corn flow velocity (0.04–0.06 m/s), air velocity (0.6–1.0 m/s), and bed thickness (20–40 cm). The pressure drop across the corn bed was measured, and the corresponding porosity was calculated using the Ergun equation. A four-factor, three-level Box–Behnken design was then applied to evaluate the effects of the selected factors and their interactions. An EDEM–Fluent coupled model was established under the corresponding operating conditions. The simulated porosity was compared with the measured and response-surface predicted values. The model was further used to analyze the local distribution of porosity within the flowing bed.

Moisture content, corn flow velocity, and bed thickness affect particle contact, loading, and rearrangement within the bed. These changes are expected to produce larger variations in dynamic porosity than the change in air velocity within the range tested in this study. The experiments and simulations were therefore conducted under matched operating conditions to test this relationship. Unlike previous work that mainly considered either a fixed grain bed or particle flow alone, the present study combines dynamic-bed measurements with EDEM–Fluent coupling under simultaneous corn flow and airflow. The comparison of measured, simulated, and predicted values provides a direct check of the coupled model. The local simulation further describes the non-uniform porosity within the moving bed. The results show that moisture content, corn flow velocity, and bed thickness have stronger effects on dynamic porosity than air velocity under the tested conditions. The coupled model also reproduces the experimental trend well. This approach provides a basis for describing bed-structure changes under operating conditions relevant to convective corn drying.

## 2. Materials and Methods

### 2.1. Test Materials

Yudan 59 corn produced in Rongchang District, Chongqing, China, was used as the test material. This variety is a hybrid of Yudan 7889 and Yudan 22. Before the experiments, corn kernels were randomly selected from the same batch, and dust and impurities were removed manually. According to kernel morphology, the selected kernels were classified into three groups: horse-tooth shape, spherical-like, and rectangular shapes [16], with a selection ratio of 1:1:1. Kernels within each group had similar sizes, uniform color, and similar fullness.

The initial moisture content of the corn samples was approximately 12% w.b. According to the experimental design, the corn samples from the same batch were divided into three batches, with target moisture contents of approximately 13%, 23%, and 33% w.b. The amount of distilled water required for each batch was calculated using the mass balance relationship for wet-basis moisture content [17]:

(1) $$W=m_{0}(\frac{M_{t}-M_{i}}{100-M_{t}})$$

where *W* is the mass of distilled water added (g), *m*_0_ is the mass of the corn sample before conditioning (g), *M_i_* is the initial moisture content (% w.b.), and *M_t_* is the target moisture content (% w.b.).

Based on the calculation, the required amount of distilled water was uniformly added to each batch, and the samples were thoroughly mixed to improve the uniformity of water distribution. The mixed samples were then sealed separately in polyethylene bags and equilibrated at 4 °C for 72 h, allowing the added water to redistribute within the kernels and among the samples [18,19].

After equilibration, representative subsamples were randomly collected from different positions of each batch, and the actual moisture content was determined in triplicate [17,18]. The moisture content of the corn was measured using the oven-drying method, with the samples dried at 103 °C for 72 h [17,19]. The wet-basis moisture content was calculated as follows:

(2) $$M=\frac{m_{w}-m_{d}}{m_{w}}\times 100\%$$

where *M* is the wet-basis moisture content (%), *m_w_* is the sample mass before drying (g), and *m_d_* is the sample mass after drying (g). The mean of the three measurements was used as the actual moisture content of each batch. The final mean moisture contents were 13.03%, 23.20%, and 33.37% w.b., respectively, and these values were used as the three moisture-content levels in the subsequent experiments.

The true density of the corn samples at each moisture level was measured using an electronic balance and a graduated cylinder, and the results are shown in Table 1.

### 2.2. Experimental Setup and Principles

**Figure 1:**
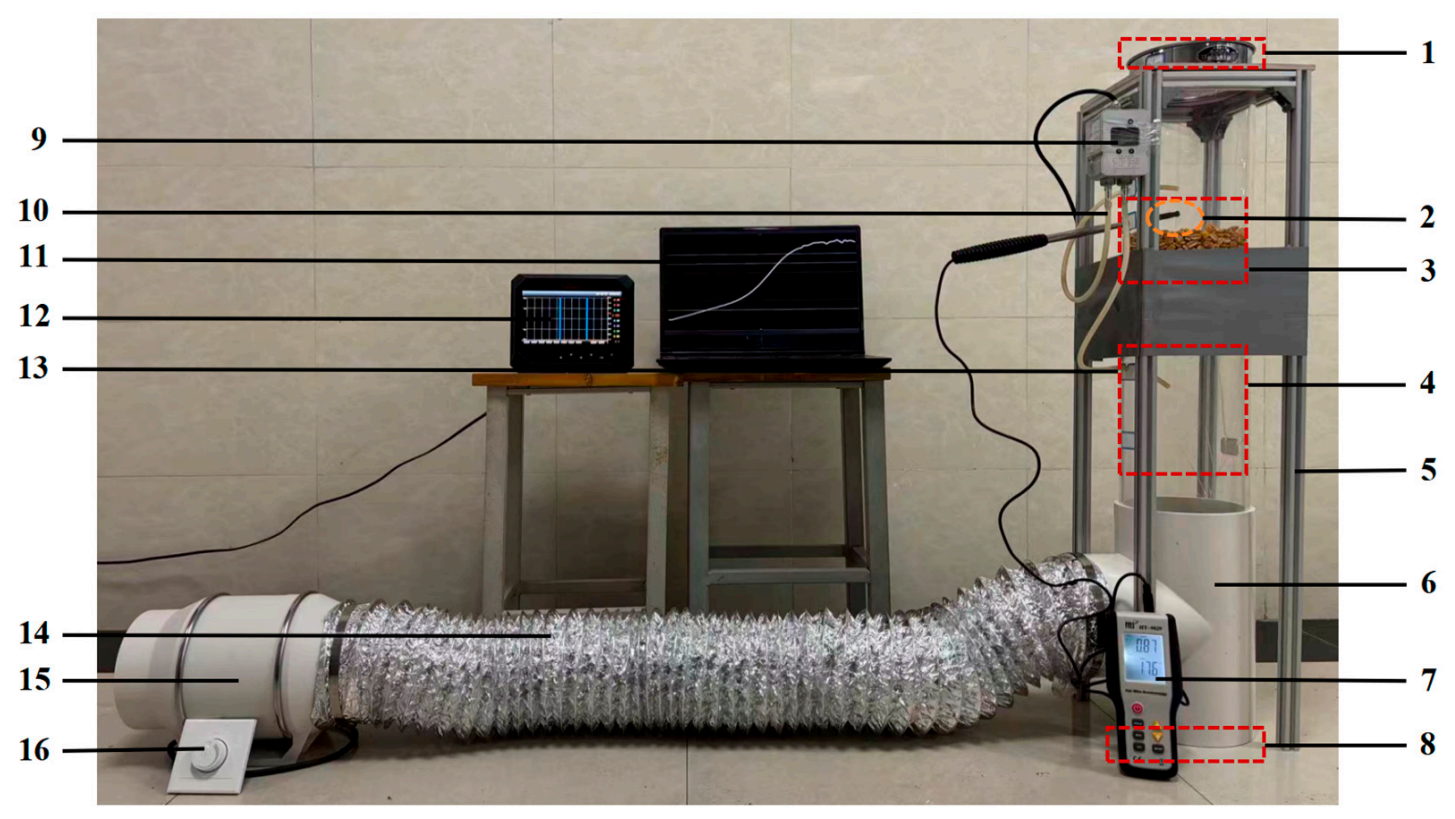
Experimental platform for determining corn-bed porosity. 1. Corn inlet; 2. air pressure sensor; 3. porosity determination zone; 4. corn–air convection zone; 5. support frame; 6. PVC tee; 7. air-velocity monitor; 8. corn outlet; 9. differential pressure monitor; 10. differential pressure monitoring tube A; 11. laptop; 12. paperless recorder; 13. differential pressure monitoring tube B; 14. ventilation duct; 15. centrifugal fan; 16. air-velocity regulator.

The experimental platform used to determine the dynamic porosity of the flowing corn bed mainly consists of a supporting structure, a variable-frequency fan, a measurement area, a fan-blade valve device, a circulation device, a convection area, a differential-pressure measurement system, and an air-velocity monitor. The physical testing platform is shown in Figure 1. It was constructed using materials such as profiles, acrylic pipes, and PVC three-way pipes, and the inner diameter of the ventilation duct *D_t_* was 150 mm. The variable-frequency fan supplies airflow to the test section through the ventilation duct and the air velocity is adjusted using the air-velocity regulator. Two pressure-monitoring tubes are connected to the differential-pressure measurement system to obtain the pressure drop across the corn bed. The platform also records the airflow velocity and these measured parameters are subsequently used to determine the bed porosity. The detailed calculation is based on the Ergun equation given as Equation (6) in Section 2.3.2.

Corn kernels are filled into the measurement area of the testing platform and the flow velocity of the corn is controlled by the opening degree of the fan-blade valve. The corn kernels enter the measurement zone from the top and move downward under gravity before leaving through the outlet. During the experiment, corn kernels with the same moisture content are continuously supplied from the top to maintain the required bed thickness. The Box–Behnken experimental scheme is implemented on the platform. The pressure drop and air velocity are recorded under each operating condition and are used for the subsequent calculation of bed porosity.

**Figure 2:**
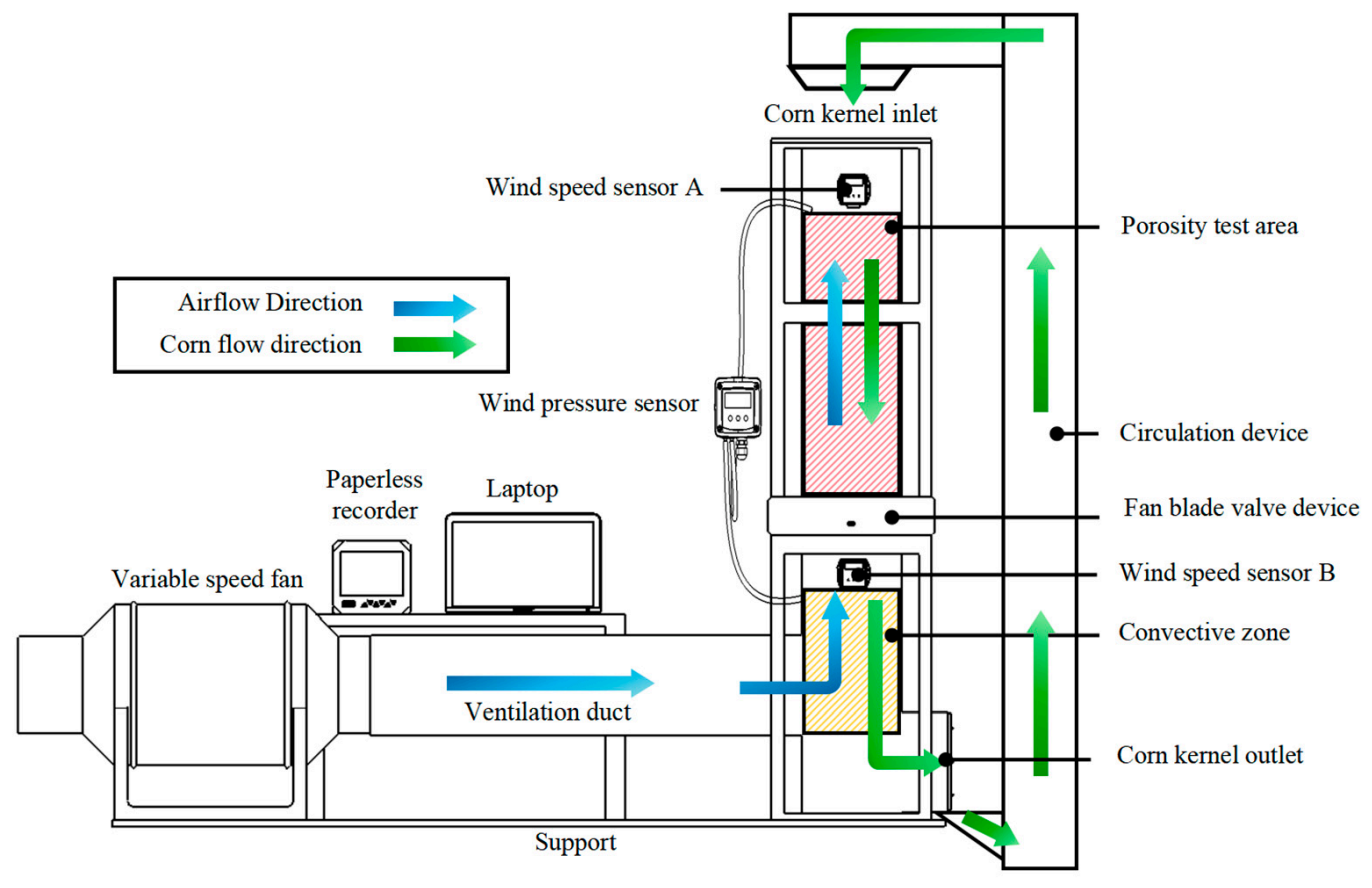
Schematic diagram of the working principle of the corn-bed porosity determination platform.

The working principle of this platform is shown in Figure 2. Corn kernels enter the measurement area through the top inlet. Under the action of gravity, they move downward through the measurement zone and the falling velocity is adjusted by the opening of the fan-blade valve. The airflow generated by the variable-frequency fan passes through the corn layer from bottom to top through the ventilation duct and is then discharged from the top. When the airflow passes through the corn layer, the inter-particle voids and particle surfaces produce flow resistance, thereby forming a pressure drop between the upstream and downstream sides of the bed layer. The differential-pressure measurement system records this pressure difference. The air-velocity sensors A and B record the airflow velocities at the corresponding positions, and the signals are recorded in real time by the paperless recorder. During the experiment, the circulation device continuously operates to maintain stable airflow conditions within the system and reduce the influence of airflow fluctuations on the recorded data.

The measured pressure drop and air velocity are used as the main experimental inputs for porosity determination. Bed thickness, the equivalent diameter of the corn kernels, air density, and air dynamic viscosity are also included in the calculation. These parameters are substituted into the Ergun equation given as Equation (2) and the corresponding bed porosity is then obtained. Therefore, the porosity values reported in this study are determined from experimentally measured airflow parameters through the Ergun-based calculation.

**Figure 3:**
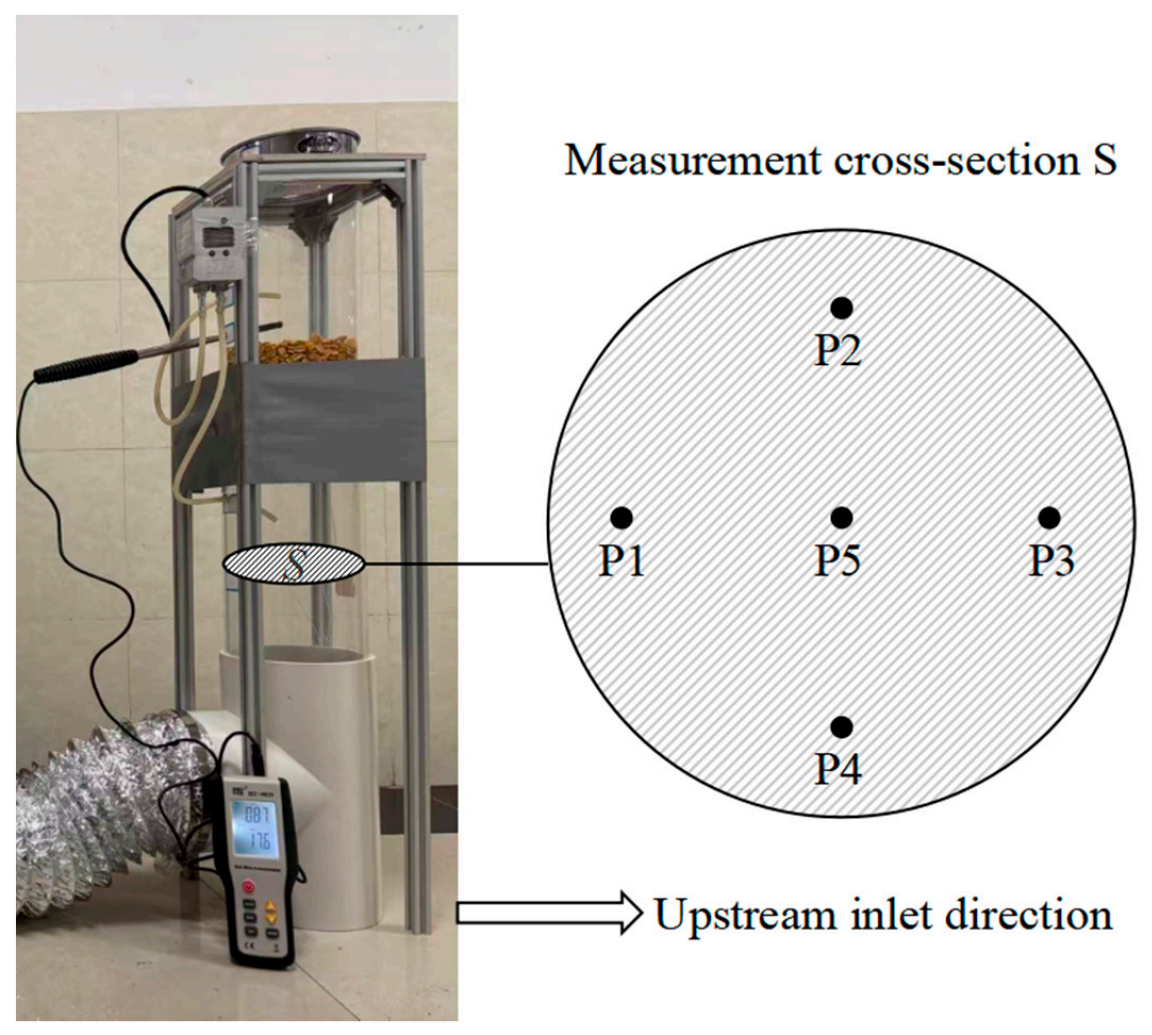
Position of measurement cross-section S and layout of air-velocity measurement points.

Considering that the upstream ventilation duct may cause a non-uniform air-velocity distribution before the airflow enters the corn bed, a measurement cross-section S was selected below the corn-bed inlet and five air-velocity measurement points P1–P5 marked by dots were arranged on the cross-section, as shown in Figure 3.. Point P5 was located at the center of the cross-section, while P1–P4 were symmetrically arranged in four directions. Before each experiment, the fan output was adjusted so that the mean air velocity across the cross-section stabilized near the target values of 0.6, 0.8, and 1.0 m/s. The coefficient of variation (*CV*) of the air velocities measured at the five points was used to evaluate the dispersion of the airflow velocity across the cross-section. After the airflow velocity became stable, the pressure drop across the corn bed and the bed porosity were measured.

To quantitatively evaluate the actual air velocity and its distribution uniformity across the measurement cross-section, the mean air velocity and the coefficient of variation (*CV*) were calculated using Equations (3) and (4), respectively:

(3) $$\bar{v}=\frac{1}{n}\sum_{i=1}^{n}v_{i}$$

(4) $$\mathrm{CV}=\frac{s}{\bar{v}}\times 100\%$$

where $\bar{v}$ is the mean air velocity across the cross-section, m/s; *v_i_* is the measured air velocity at the *i*-th measurement point, m/s; *n* is the number of measurement points, *n* = 5 in this study; *σ* is the standard deviation of the air velocities measured at the five points; and *CV* is the coefficient of variation of air velocity, %. A smaller *CV* indicates a lower dispersion among the measured air velocities and a more uniform airflow distribution across the cross-section.

### 2.3. Experimental Design and Methods

#### 2.3.1. Porous Media and Porosity

The accumulation layer is composed of corn particles and the inter-particle connecting pores. Its medium structure belongs to a typical granular loose porous medium [16]. When fluid flows within the medium, it is affected by factors such as pore structure, particle shape, and arrangement. Air mainly seeps through the inter-particle gaps, and its ventilation resistance and local pressure drop are all related to the pore structure [17]. Therefore, the corn accumulation layer can be regarded as a porous medium, which is helpful for analyzing its ventilation characteristics and establishing a coupled model.

Porosity essentially reflects the proportion relationship of the particle entities in the overall space. The higher the porosity, the more voids there are within the bed layer and the larger the flow space for the gas when passing through the corn pile; the lower the porosity, the more compact the corn pile is and the bed layer resistance is usually greater. The porosity of the corn flow layer [18] can be calculated by Formula (5):

(5) $$\varepsilon =(1-\frac{M}{V\rho_{t}})\times 100\%$$

In the above equation, ***ε***: porosity of the flow layer, %; *M*: mass of corn in the measurement area, g; *V*: volume of the measurement area, cm^3^; ***ρ_t_***: true density of corn, g/cm^3^.

#### 2.3.2. Theoretical Calculation of Bed Porosity

This paper designs a device for measuring bed porosity and establishes a relationship between bed pressure drop and porosity based on the Ergun ventilation resistance equation, which is used to calculate the porosity of the flowing layer. The Ergun ventilation resistance equation is an important equation in fluid mechanics, used to describe fluid flow and pressure loss in particulate beds [11]. In this study, bed porosity is calculated using the Ergun ventilation resistance equation, as shown in Equation (6):

(6) $$\frac{\Delta p}{L}=\frac{150\mu {\left(1-\varepsilon \right)}^{2}}{D_{p}^{2}\varepsilon^{3}}u+\frac{1.75\rho (1-\varepsilon )}{D_{p}\varepsilon^{3}}u^{2}$$

In the above equation, ∆*p*: pressure drop of the corn grain layer, Pa; ***L***: thickness of the drying layer, m; ***D_p_***: equivalent diameter of the corn, m; ***ε***: porosity of the flow layer, %; ***u***: air flow velocity in the measurement area, m/s; ***μ***: dynamic viscosity of air, Pa·s; ***ρ***: air density, kg/m. Among them, the equivalent diameter ***D_p_*** of the corn [20] can be calculated by the following formula:

(7) $$D_{p}=\frac{3L_{0}\mathrm{BH}}{L_{0}H+\mathrm{HB}+L_{0}B}$$

In the formula, *L*_0_, *B*, and *H* represent the average values of the length, width, and height of the corn, respectively, in millimeters.

Different moisture contents of corn were prepared and measured on the porosity testing platform according to the experimental scheme in Table 2. The grain layer resistance and the air flow speed in the test chamber were tested. The porosity of the corn was calculated by combining Equations (6) and (7). The air parameters in Equation (6) are shown in Table 2. The ventilation resistance of the flow layer of corn with different moisture contents was tested using the air parameters [21] in Table 2.

#### 2.3.3. Theoretical Assumptions

To simplify the theoretical analysis of the dynamic porosity of the corn bed, the following assumptions were made:

(1) At the macroscopic scale of the measurement region, the dynamic corn bed was treated as a granular porous medium and its pore structure was characterized by the volume-averaged porosity [22].
(2) Within the time scale considered in this study, the corn kernels were assumed to undergo no significant deformation or breakage. In the theoretical calculation based on the Ergun equation, the equivalent particle diameter *D_p_* was used to represent the characteristic particle size, whereas the non-spherical corn kernels were still modeled using the multi-sphere method in the EDEM simulation [23].
(3) The porosity, air velocity, and pressure drop across the corn bed were represented by their macroscopic average values within the measurement region. The relationship between pressure drop and bed porosity was established using the Ergun model [24,25].
(4) Wall effects were assumed to be mainly confined to the near-wall region. In this study, the ratio of the inner diameter of the measurement cylinder to the equivalent particle diameter (*D_t_*/*D_p_*) was used to characterize the relative scale between the bed and the particles. Under the present geometric conditions, the influence of wall effects on the overall bed characteristics was considered relatively weak. Therefore, no separate correction for wall effects was applied [26,27,28].
(5) Under each operating condition, the moisture content, dimensions, and mass of an individual corn kernel were assumed to remain constant. Changes in particle mass and dimensions caused by moisture evaporation during the short-term airflow process were neglected. The influence of airflow on particle motion was represented through gas–solid coupling.
(6) During each measurement, corn kernels were continuously supplied to maintain an approximately constant bed thickness. Therefore, the bed thickness L was treated as a constant parameter under a given operating condition.

#### 2.3.4. Analysis of Airflow Velocity in the Corn Bed

**Figure 4:**
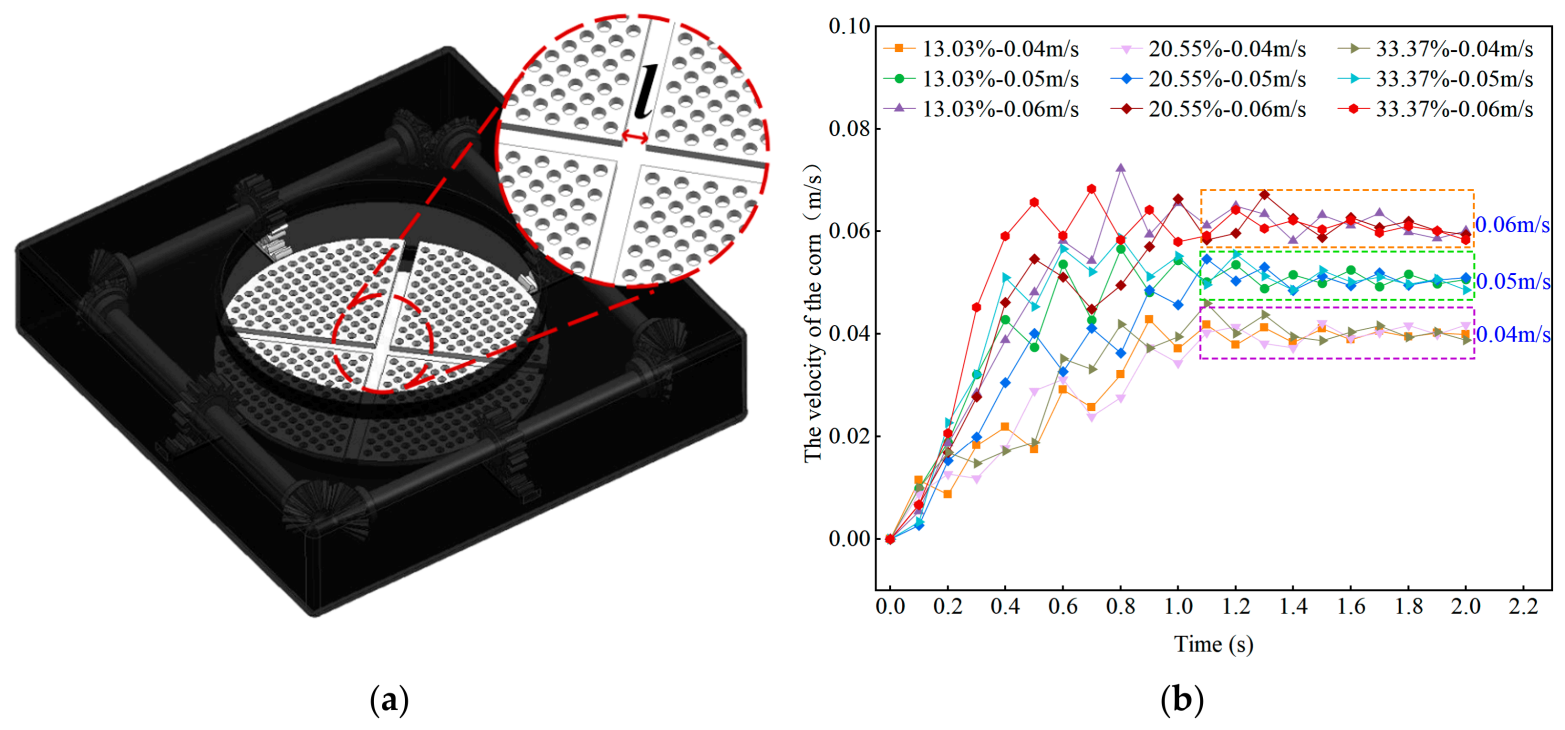
Corn flow rate regulating mechanism (**a**) and corn flow rate diagram (**b**).

This study regulates the valve opening degree *l* by controlling the relative distance of the fan blades, thereby achieving the adjustment of the laminar flow velocity of the corn through the valve opening degree *l*. The schematic diagram of the fan blade valve is shown in Figure 4a. The relationship between the valve opening degree *l* and the corn flow rate was experimentally tested and obtained, as shown in Figure 4b. Fill the required corn in the porosity test layer. When the valve is opened, manually fill the same moisture content corn particles to keep the thickness of the corn in the test layer unchanged. The corn in the porosity test layer flows downward, and the falling corn mass is recorded by the paperless recorder and brought into Equation (8) to calculate the corn flow rate.

(8) $$v=\frac{m}{\mathrm{\rho S}}$$

*m*: mass flowing through per unit time (in kilograms); *ρ*: density of corn, kg/m^3^; *S*: flowing cross-sectional area, m^2^; *v*: Flow velocity of corn per unit time, m/s.

The corn flow rate obtained from Equation (5) is shown in Figure 4b. During the period of 0–1.1 s, the corn flow rate fluctuated significantly. It is speculated that, during this period, the fan blade valve was opened, causing the corn to suddenly change from a stationary state to a moving state. Additionally, the friction coefficients between the corn and the fan blade valve were relatively high, which led to unstable movement speed within the porosity measurement area and significant jitter. During the period of 1.1–2 s, the movement speed of the corn particles in the porosity test layer tended to be stable, with smaller speed fluctuations. When the moisture content of the corn was 13.03% w.b., 23.2% w.b., and 33.37% w.b., the corresponding stable flow speed of the valve opening was 0.04 m/s, 0.05 m/s, and 0.06 m/s, respectively. The relationship between the valve opening and the corn flow rate is shown in Table 3.

### 2.4. Establishment of a Bed Porosity Simulation Measurement Model

#### 2.4.1. Establishment of the Corn Particle Model and Platform

**Figure 5:**
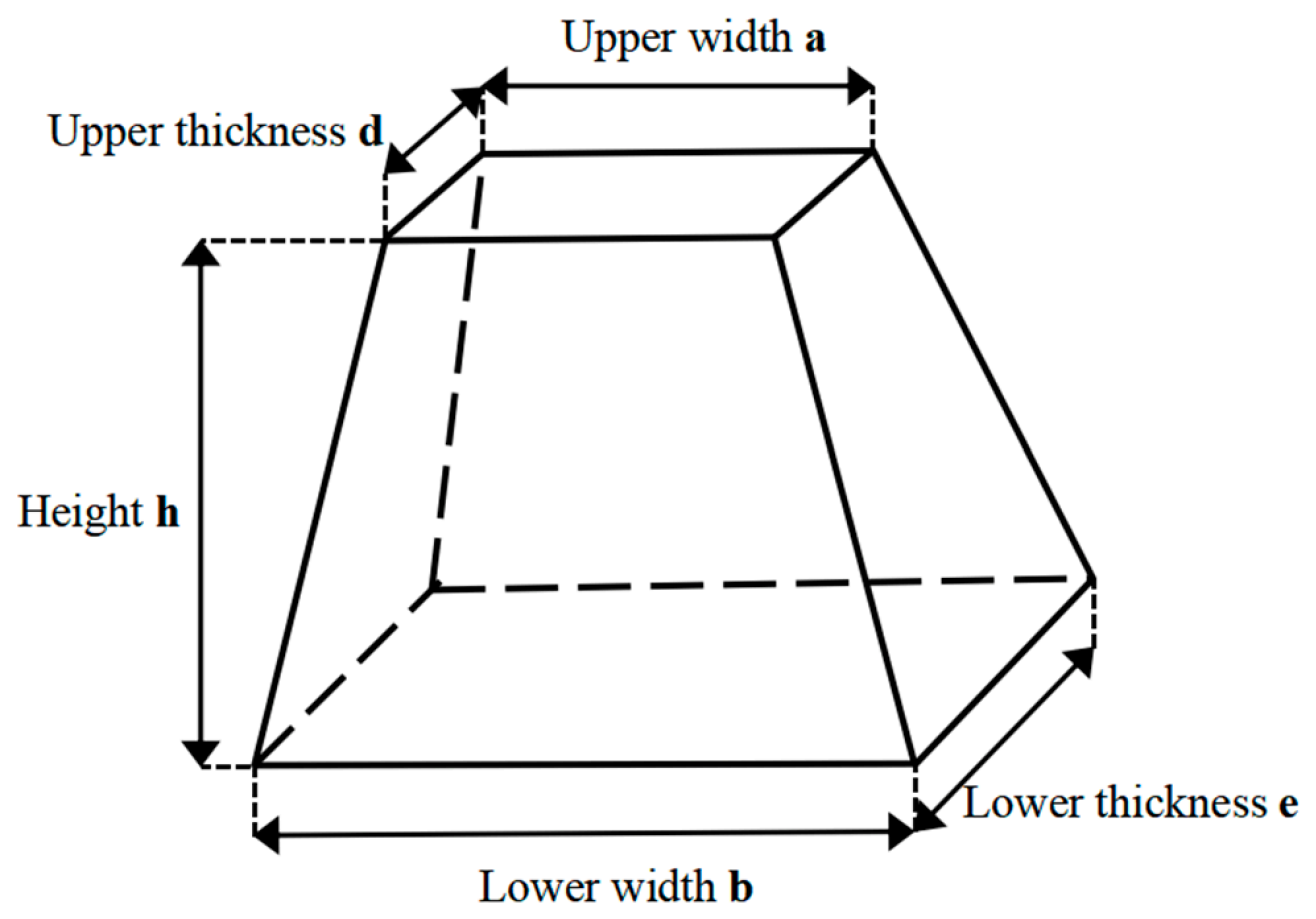
Illustration of the shape of corn.

The corn particle samples were classified into horse-tooth shape, spherical-like and rectangle shapes [23]. The three-dimensional geometric dimensions of the corn particles were measured using a vernier caliper. As shown in Figure 5, the three-dimensional geometric dimensions of the corn particles can be represented by length, width and height. One hundred particles of each of the three shapes were selected, the external dimensions of the three different shapes of particles were measured using a vernier caliper with an accuracy of 0.01 mm, and the average values were calculated as shown in Table 4.

**Figure 6:**
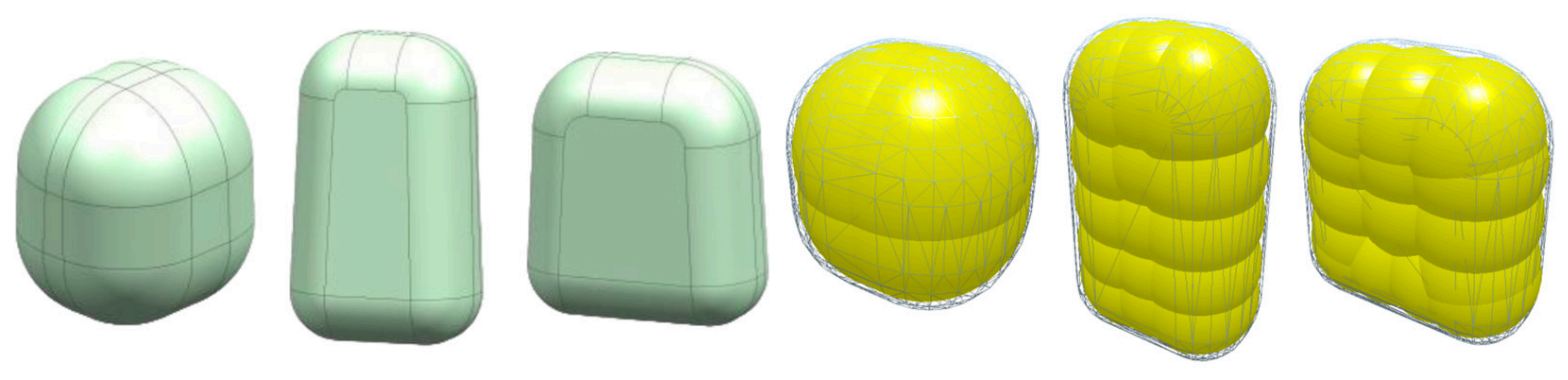
Rectangular particle geometric model and sphere filling.

Based on the measured average size of the corn particles, using UG software, geometric models were respectively established for three types of corn particles: spherical, horse-tooth shape, and rectangular. After the modeling was completed, stl files were output and imported into the particle template of EDEM software for sphere filling. The filling situation is shown in Figure 6. In EDEM software, the filling of the template model can be achieved by setting the number, coordinates, and diameter of the spheres. Generally, the more filling spheres there are, the higher the geometric accuracy of the particle model will be, but it will also occupy more computing memory. Therefore, when establishing the particle model and filling the spheres, the simulation accuracy requirements should be considered based on the computer hardware configuration to avoid operation lag or even freezing due to excessive computing load. Therefore, based on the actual situation of the research, this paper fills 4 spheres for spherical particles, 15 spheres for horse-tooth shape particles, and 15 spheres for rectangular particles. The filling effects of the three types of corn particles are restored to the actual appearance characteristics of corn and also reduce the experimental error.

**Figure 7:**
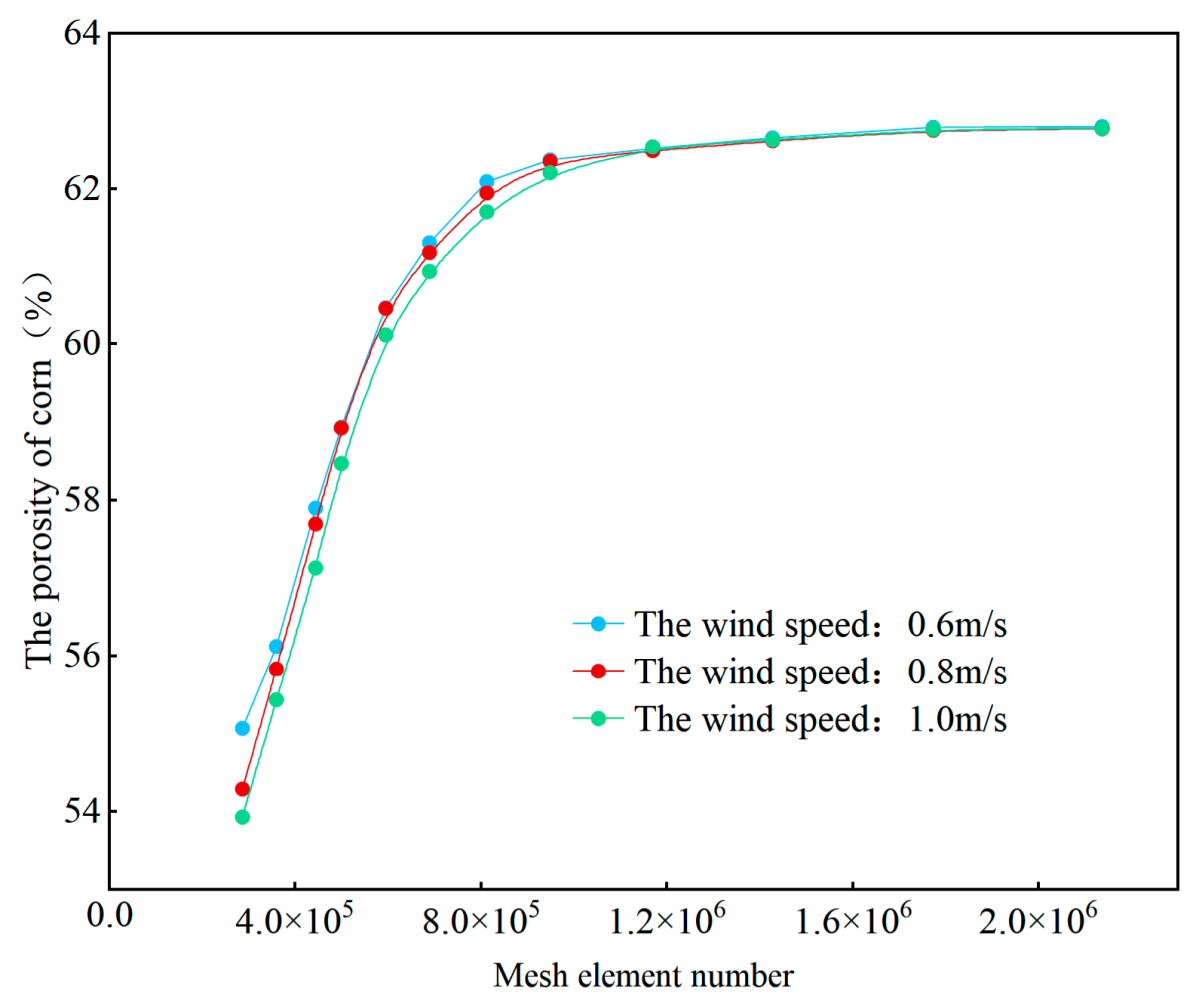
Mesh independency analysis.

The quality of the grid structure has a significant impact on the accuracy of the numerical simulation results. Usually, it can be evaluated by combining indicators such as skewness and result stability, thereby selecting the optimal grid structure in the simulation model to ensure the accuracy and stability of the porosity calculation results [29]. As shown in Figure 7, the results of the grid independence analysis of the model structure are presented. It can be observed that, after point A, the simulated porosity values of each state tend to stabilize. Therefore, the grid structure at point A is selected for the simulation. The relevant evaluation values are detailed in Table 5.

**Figure 8:**
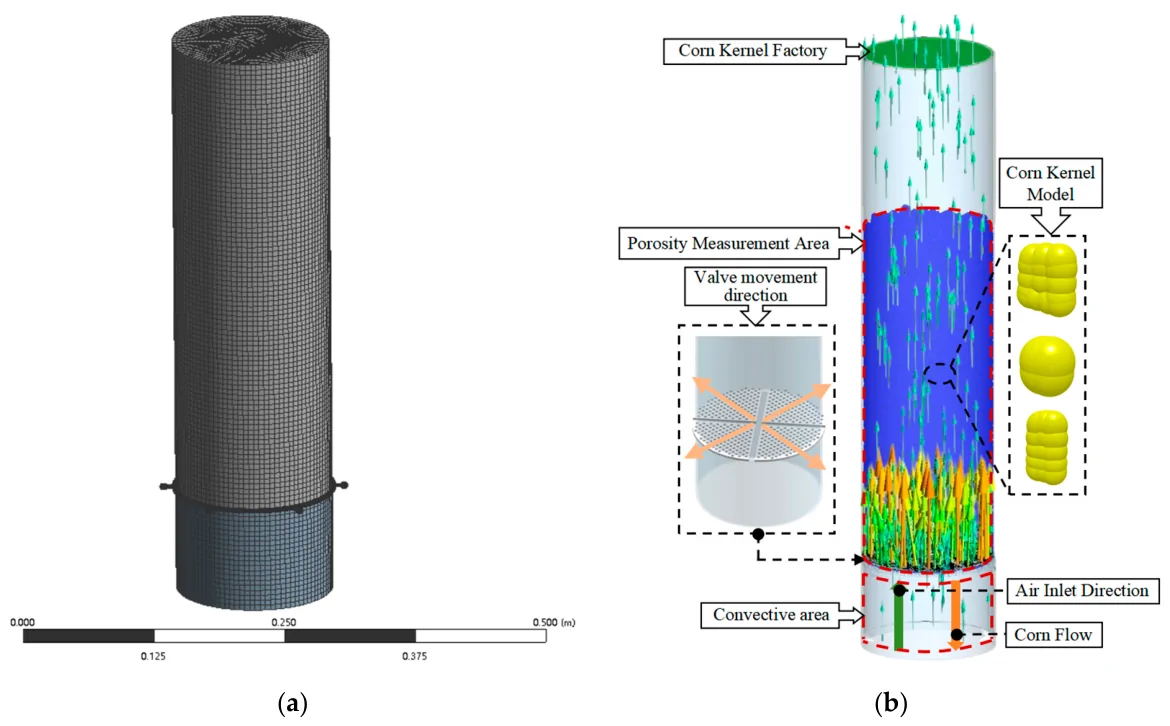
Grid division of the porosity measurement device (**a**) and simulation model (**b**).

To reduce the computational cost, the numerical model was simplified. The upstream ventilation duct of the porosity measurement device contains a bend, while the present simulation mainly focuses on the gas–solid coupling behavior and pore-structure changes within the dynamic corn bed. Therefore, the complete upstream ventilation duct was not included in the computational domain. Accordingly, a simplified geometric model of the porosity measurement device was established and imported into Fluent for meshing, as shown in Figure 8a. The corn material properties and contact parameters used in the numerical simulation are listed in Table 6. The parameter values were determined based on the ranges reported in previous studies [30], and the reasonableness of the parameter settings was assessed based on the consistency between the simulation results and the experimental data. In the EDEM software, a corn particle model factory was constructed, and the corn particles were filled in accordance with the sampling ratio of the three corn shapes of 1:1:1 to generate a corn layer with a certain thickness formed by the accumulation of several corn particle models. The simulation controlled the flow velocity of the corn particle models by adjusting the opening degree of the fan blade valve. During the simulation process, the airflow entered the convective area from the bottom (the corn moved downward, and the airflow moved upward), passed through the fan blade valve, entered the corn layer in the porosity test area, and finally flowed out from the top of the corn layer. The bed porosity measurement simulation model is shown in Figure 8b.

#### 2.4.2. The Variation Characteristics of Corn Speed in the Simulated Measurement Layer of Bed Porosity

**Figure 9:**
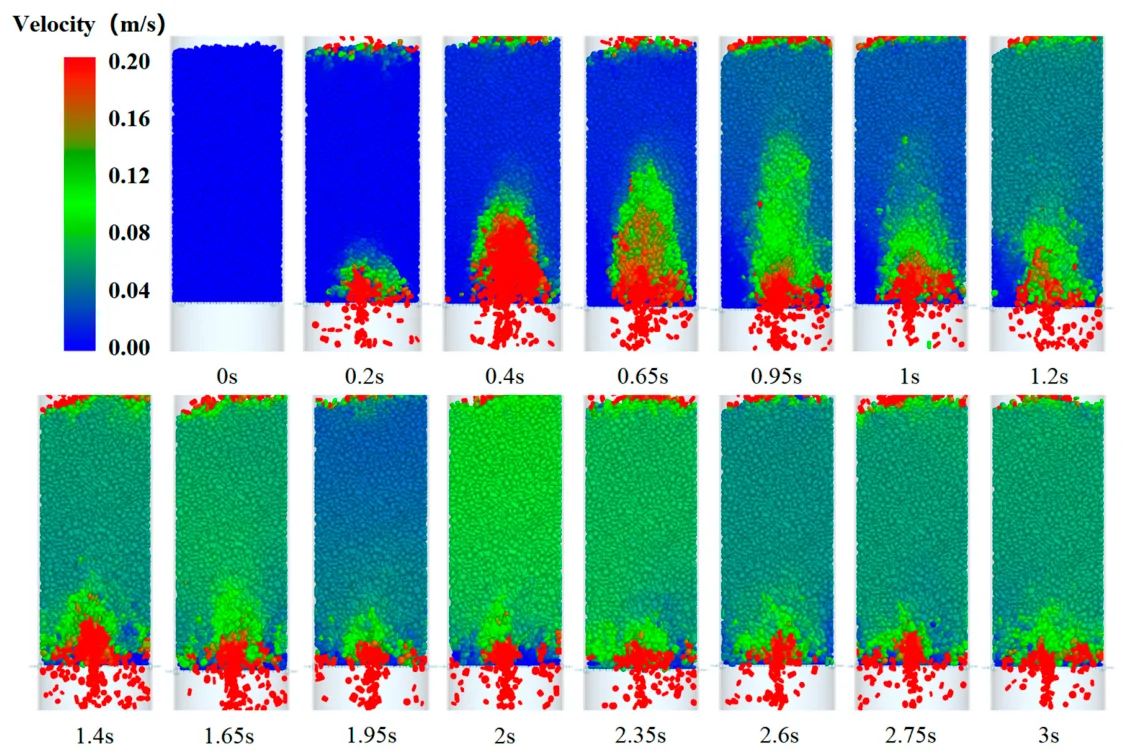
Corn flow velocity variation diagram.

To investigate the movement state of corn within the pores of the layer from unstable to stable, the process was simulated using the EDEM–Fluent coupling simulation, as shown in Figure 9. As can be seen from the figure, the velocity field of the dynamic layer of corn evolves over time into three stages: initiation, development, and stability. In the 0–0.4 s stage, the valve opening gradually increases and the bottom corn particles show a slight “bridging”. Under the action of the airflow, the corn particles begin to undergo significant rolling and upward movement. In the 0.65–1.65 s stage, the “bridged” corn particles collapse, and the bottom corn particles show a high-speed downward falling trend and continuous upward expansion. The main body of the bed layer gradually changes from low speed to medium speed, and the dynamic layer range expands rapidly. At this time, a clear “lower high and upper low” flow velocity gradient characteristic is formed within the bed layer. In the 1.95–3 s stage, the overall speed distribution of the bed layer tends to be uniform, while only a local area at the bottom still maintains a relatively high speed fluctuation, indicating that the corn dynamic layer has gradually transitioned from the initial local disturbance to a relatively stable flow state, and the system has basically reached dynamic equilibrium.

Overall, the particle movement in the corn dynamic layer exhibits a distinct temporal evolution pattern, namely, it starts from the bottom, the speed expands upward, and eventually stabilizes. Among them, the bottom area is always the region with the most intense particle movement and the most active area for bed layer structure changes. This indicates that the velocity distribution within the corn dynamic layer has significant spatial non-uniformity, and this feature will directly affect the evolution pattern of the bed layer porosity.

### 2.5. Experimental Scheme

In order to explore the variation pattern of corn-bed porosity, after reviewing relevant literature, this study found that the main factors affecting the porosity of granular crops include moisture content, air velocity, temperature, bed height, and material type [9]. Considering the objective of this study and the feasibility of the experiments, four factors were selected: moisture content, corn flow rate, air velocity, and bed height. Preliminary single-factor experiments were first conducted to examine the individual effect of each factor on corn-bed porosity. The experimental scheme is shown in Table 7. These experiments were used as preliminary tests and provided a basis for setting the factor levels in the subsequent response surface experiment.

Corn-bed porosity (*Y*) was selected as the response variable. A four-factor, three-level Box–Behnken design was established using Design-Expert 13 to analyze the effects of moisture content (*A*), corn flow rate (*B*), air velocity (*C*), and bed height (*D*) on corn-bed porosity. The factor levels used in the Box–Behnken design are shown in Table 8. The design consisted of 29 experimental runs, including 24 non-center runs and five replicated center-point runs. The center-point condition was *A* = 23.20% w.b., *B* = 0.05 m/s, *C* = 0.8 m/s, and *D* = 30 cm. The five center-point runs were performed as independent experimental replicates under the same factor settings, rather than as repeated measurements within a single experimental run. One corn-bed porosity value was obtained from each independent replicate. Corn samples conditioned to 23.20% w.b. as described in Section 2.1 were used for the center-point runs. The response values obtained from all 29 runs were entered separately into Design-Expert 13 for regression analysis and ANOVA, with the five center-point responses used to estimate pure error. When the corn-bed heights were 20, 30, and 40 cm, the corresponding test-chamber volumes were 3534.29, 5301.44, and 7068.58 cm^3^, respectively. After the response surface model was established using these 29 experimental runs, five additional independent validation experiments were conducted. The complete experimental matrix and measured responses can be found in Section 3, Results and Discussion. Moreover, this section also presents the comparison between predicted values and measured porosity from the validation experiments.

### 2.6. EDEM–Fluent Coupling Simulation Analysis

**Figure 10:**
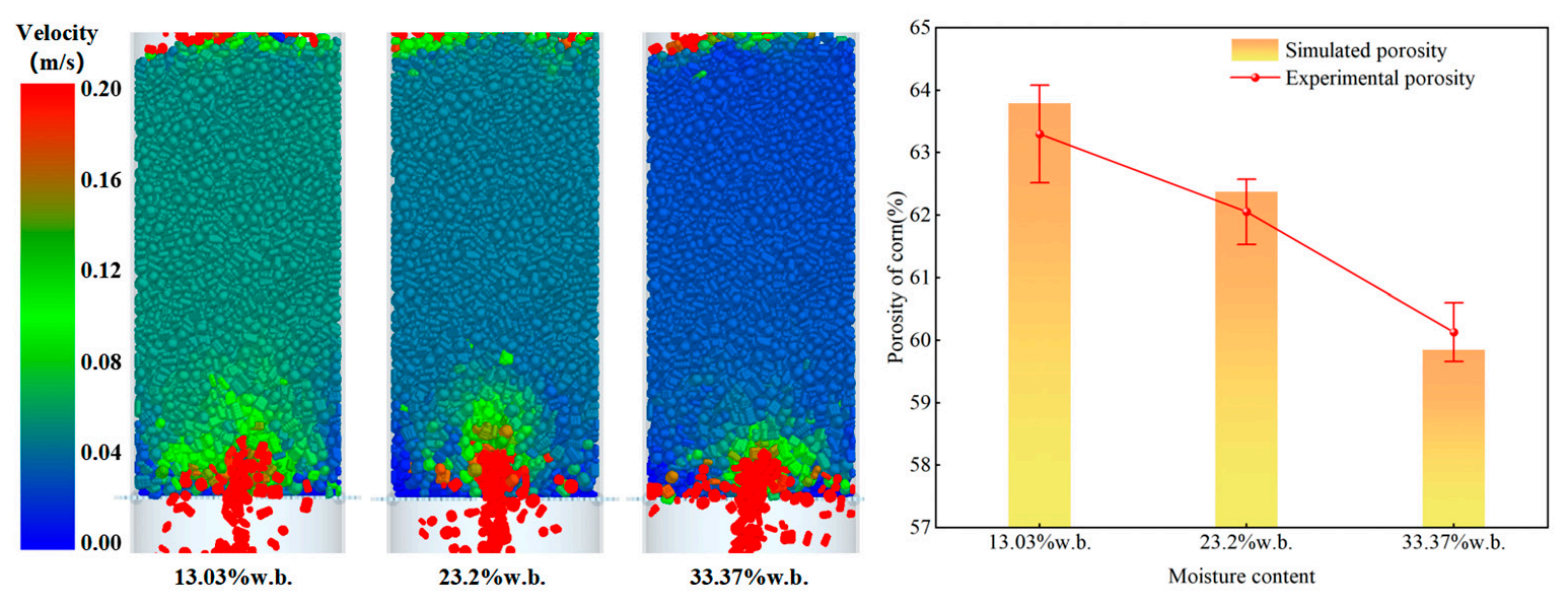
Effect of *M_c_* on porosity *ε*.

**Figure 11:**
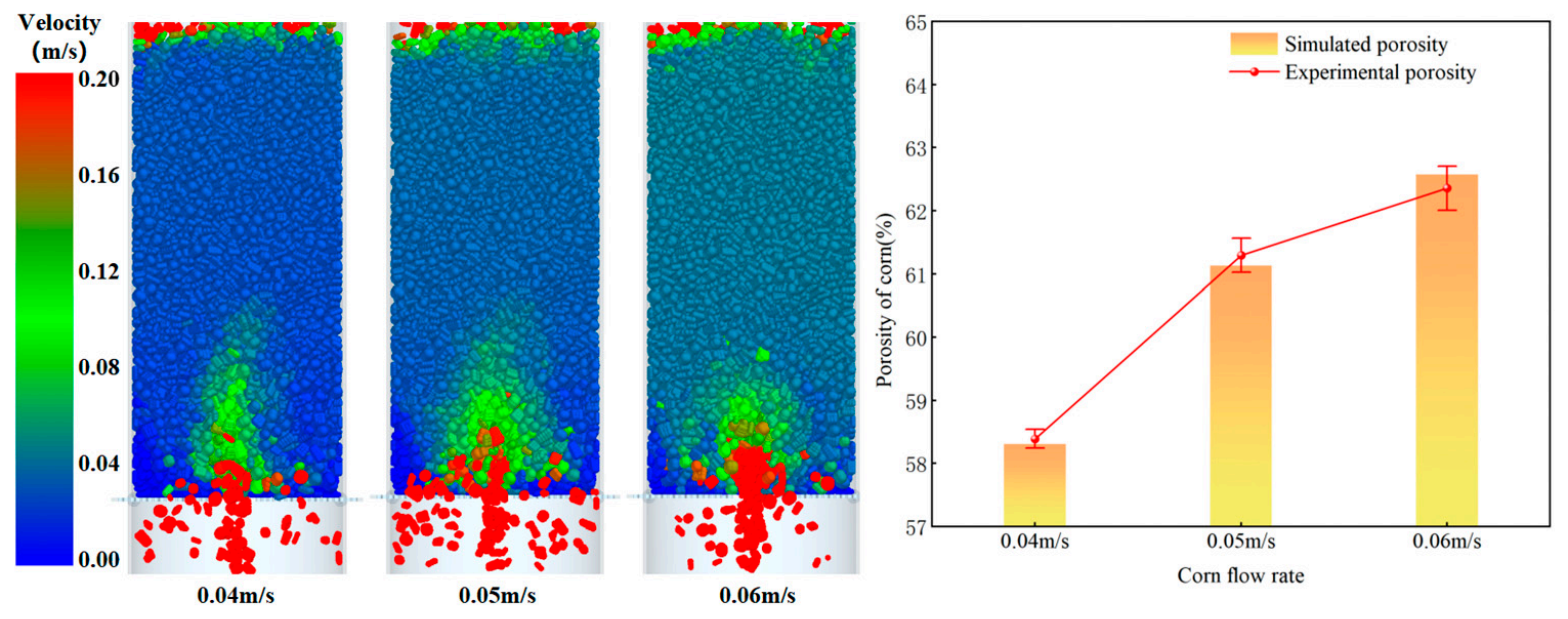
Effect of *V_d_* on porosity *ε*.

**Figure 12:**
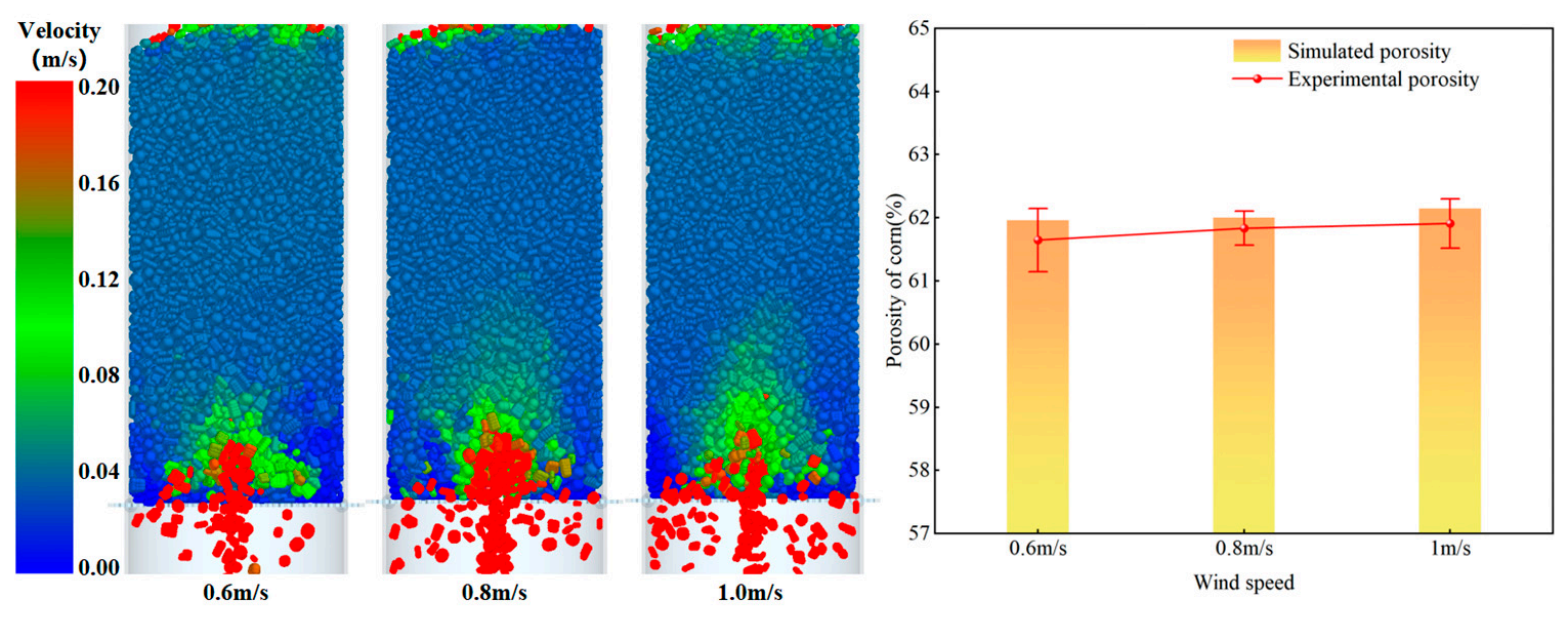
Effect of *V_W_* on porosity *ε*.

**Figure 13:**
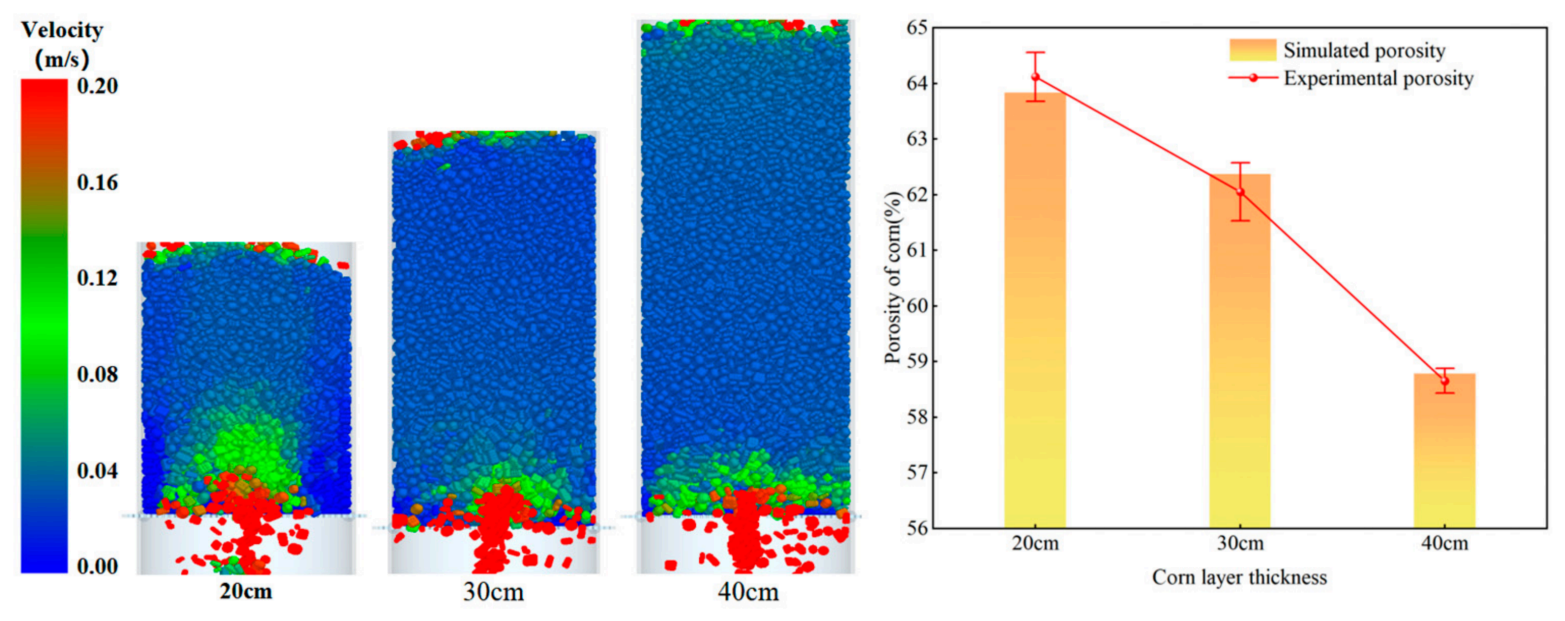
Effect of *H* on porosity *ε*.

To deeply explore the extent to which various factors affect the porosity during the experiment, based on the single-factor experimental design in Table 7, the coupling simulation results of EDEM–Fluent were compared with the measured porosity. Figure 10 shows that, as the moisture content *M_c_* increases, the porosity of the dynamic corn layer continuously decreases, indicating that moisture content has a significant impact on porosity. This trend may be related to changes in the contact behavior of corn kernels as moisture content increases, which may promote a denser packing structure in the bed. In Figure 11, when the corn flow rate *V_d_* increases, the porosity significantly increases, indicating that when the bed layer movement strengthens, the particle rolling and rearrangement become more intense, and the bed layer structure becomes more loose. Figure 12 shows that the change in air speed *V_w_* has a weak effect on porosity, causing only minor fluctuations within the set working conditions, indicating that air speed is not the main controlling factor for porosity changes under these conditions. Figure 13 indicates that, when the layer thickness *H* increases, the porosity decreases synchronously, suggesting that a larger material layer thickness will strengthen the bed layer compaction effect, thereby compressing the pore space between particles.

Overall, the dynamic porosity of the corn layer is mainly influenced by the moisture content of the corn particles, the corn flow rate, and the thickness of the material layer. Among them, the moisture content and the layer thickness are negative factors, while the corn bed speed is a positive factor and the effect of air speed is relatively limited.

### 2.7. Experimental and Model Uncertainty Analysis

Uncertainty analysis is an important approach for evaluating the reliability of experimental measurements and numerical results [31]. For the dynamic porosity of corn beds, both measurement errors and model parameters may contribute to uncertainty. Since the corn bed undergoes continuous rolling, sliding, collision, and rearrangement under airflow, its internal structure exhibits significant dynamic characteristics. Consequently, measurement errors in parameters such as outlet air velocity, pressure drop, bed velocity, valve opening, volumetric flow rate, and equivalent diameter of corn particles may all influence the calculated porosity values. Uncertainty analysis enables quantitative assessment of the variability among measured parameters and provides a basis for evaluating the credibility of subsequent porosity results.

This study uses the repeated measurement statistical method to evaluate the uncertainty of the main test parameters. For the data obtained by repeated measurement r times under the same working condition, the average value is as follows:

(9) $$\bar{N}=\sum_{i=1}^{r}N_{i}/r$$

In the formula: $\bar{N}$ represents the average measured value of the parameter; ***N_i_*** represents the measurement value of the *i*-th time; ***r*** represents the number of repeated measurements.

The uncertainty of the parameter is expressed by the degree of dispersion of the repeated measurement values relative to the average value, that is,

(10) $$u=\sqrt{\sum_{i=1}^{r}{\left(N_{i}-\bar{N}\right)}^{2}/r(r-1)}$$

In the formula, *u* represents the standard uncertainty of this parameter.

The above method mainly reflects the random fluctuations in the repeated measurement process and is applicable to the statistical analysis of dynamic parameters such as outlet air speed, air pressure difference, and corn-bed speed. For parameters such as valve opening degree, volumetric flow rate, and corn equivalent diameter, their uncertainties are determined based on the repeated measurement results, combined with instrument resolution, reading accuracy, and calculation errors. According to the accuracy of the test device, the stability of the measurement process, and the movement characteristics of corn dynamic layer particles, the uncertainty results of the main test parameters in this paper are shown in Table 9.

Table 9 summarizes the uncertainties of the main experimental parameters and their sources. The uncertainty of outlet airflow velocity is mainly associated with velocity measurement and flow-field fluctuations. The uncertainty of pressure drop is related to the accuracy of the differential-pressure sensor and transient pressure variations across the corn bed. The uncertainty of corn-bed velocity mainly arises from fluctuations in particle motion and the calculation of flow velocity. The uncertainty of valve opening is mainly associated with mechanical adjustment and reading errors, while the uncertainty of equivalent particle diameter is related to dimensional measurement and differences among individual kernels.

The values in Table 9 characterize the uncertainty of each measured quantity separately. To evaluate their combined contribution to the final dynamic porosity, a sensitivity-based uncertainty propagation method was used to calculate the combined standard uncertainty of porosity.

The propagation model followed the porosity calculation procedure used in this study. Pressure drop ∆*P*, airflow velocity *V_w_*, and equivalent particle diameter *D_p_* are input quantities in the Ergun equation given in Equation (6), and their uncertainties were propagated through the corresponding porosity calculation. Bed depth *L*, air density *ρ*, and dynamic viscosity *μ* are also included in Equation (6). In the propagation calculation, *L* was specified according to each experimental condition, while *ρ* and *μ* were taken from Table 2. Because Table 9 does not provide separate standard uncertainties for *L*, *ρ*, and *μ*, these quantities were treated as fixed inputs.

For the uncertainty propagation analysis, the equivalent particle diameter was calculated using Equation (7) and the kernel dimensions listed in Table 4. For each kernel shape, *L*_0_ = *h*, *B* = (*a + b*)/2, and *H* = (*d* + *e*)/2. The equivalent diameters of the spherical-like, horse-tooth, and rectangular kernels were 8.7333, 6.9029, and 7.3102 mm, respectively. According to the 1:1:1 sampling ratio of the three kernel shapes, the mean equivalent particle diameter used in the propagation calculation was *D_p_* = 7.6488 mm.

Corn-bed velocity *V_d_* characterizes the particle-flow state within the measurement region. Changes in *V_d_* affect particle rolling, collision, and rearrangement, and consequently change the bed structure. Equation (7) includes *V_d_* as factor *B*, so its uncertainty contribution was evaluated from the local sensitivity of the response-surface model to *B*. Valve opening *l* and volumetric flow rate *Q* characterize the operating and flow-measurement conditions of the experimental system. The propagation calculation was formulated using ∆*P*, *V_w_*, *V_d_*, and *D_p_* as the main uncertain inputs. The effects associated with valve opening and volumetric flow rate were represented through the measured corn-bed velocity and airflow condition, respectively. For an input quantity *x_i_*, the corresponding sensitivity coefficient is as shown in Equation (11). For pressure drop, airflow velocity, and equivalent particle diameter, the sensitivity coefficients were obtained from the implicit form of Equation (6), as shown in Equation (12).

(11) $$C_{i}=\frac{\partial Y}{\partial x_{i}}$$

(12) $$F(\varepsilon )=\frac{\Delta P}{L}-\frac{150\mu V_{w}{\left(1-\varepsilon \right)}^{2}}{D_{p}^{2}\varepsilon^{3}}-\frac{1.75\rho V_{w}^{2}(1-\varepsilon )}{D_{p}\varepsilon^{3}}=0$$

Accordingly, the sensitivity coefficient of porosity to an input quantity *x_i_* is expressed as

(13) $$C_{i}=\frac{\partial Y}{\partial x_{i}}=-100\frac{\partial F/\partial x_{i}}{\partial F/\partial \varepsilon }$$

For corn-bed velocity, the sensitivity coefficient derived from Equation (14) is

(14) $$C_{V_{d}}=-125.30285+8.21632A-62.08750C+7.91100D-3222.8B$$

where *A*, *B*, *C*, and *D* represent moisture content, corn-bed velocity, airflow velocity, and bed depth, respectively.

Assuming that the input quantities included in the propagation analysis were independent, the combined standard uncertainty of dynamic porosity is calculated as

(15) $$u_{c}(Y)=\sqrt{{\left(c_{\Delta P}u_{\Delta P}\right)}^{2}+{\left(c_{V_{w}}u_{V_{w}}\right)}^{2}+{\left(c_{V_{d}}u_{V_{d}}\right)}^{2}+{\left(c_{D_{p}}u_{D_{p}}\right)}^{2}}$$

where *u_i_* is the standard uncertainty of the corresponding input quantity listed in Table 9 and *c_i_* is its sensitivity coefficient. The relative combined standard uncertainty is calculated as

(16) $$u_{c,r}=\frac{u_{c}(Y)}{Y}\times 100\%$$

In addition to experimental measurement uncertainty, the particle–particle and particle–wall contact parameters may also introduce uncertainty into the DEM simulation. The restitution coefficient, static friction coefficient, and rolling friction coefficient affect particle collision, sliding, rolling, and rearrangement. Variations in these parameters may therefore change the packing state and predicted porosity of the corn bed. The agreement between the simulated and experimental results indicates that the selected parameter settings are reasonable under the present operating conditions. However, parameter-related uncertainty should still be considered when the model is applied to different corn materials or operating conditions.

## 3. Results and Discussion

In research on the flow of corn, heat transfer during drying, and moisture transfer during the drying process, Chen Zhujun [11] et al. regarded the corn layer as a porous medium and used the Ergun equation to describe the static ventilation resistance of hot air drying. The results showed that the resistance had a quadratic growth relationship with the air speed and a linear positive correlation with the thickness of the corn layer. Pressure drop serves as an auxiliary indicator of changes in the pore structure of the dynamic corn bed and is jointly affected by air velocity, bed thickness, and pore structure. An increase in air velocity or bed thickness leads to a higher pressure drop. Under the same air velocity and bed thickness, a lower porosity indicates a more compact bed structure, resulting in greater ventilation resistance and a higher pressure drop. Therefore, the characteristics of the dynamic porosity change in the corn layer revealed in this study are of great significance for research on optimizing and improving the structure of corn drying equipment. These results can provide a reference for parameter setting in subsequent models of airflow, and heat and mass transfer in corn beds, as well as for the optimization of drying equipment.

### 3.1. Analysis of Inlet Air-Velocity Uniformity

As shown in Table 10, when the target inlet air velocities were 0.6, 0.8, and 1.0 m/s, the measured mean air velocities across measurement cross-section S were 0.596, 0.802, and 1.032 m/s, respectively. The corresponding relative deviations from the target values were −0.67%, 0.25%, and 3.20%, indicating good agreement between the measured mean air velocities and the target operating conditions. The coefficients of variation (*CVs*) of air velocity under the three conditions were 4.38%, 5.17%, and 7.23%, respectively. The variation in air velocity among the measurement points was generally small, indicating that no obvious local airflow maldistribution occurred before the air entered the corn bed. Therefore, the airflow velocity distribution across the measurement cross-section was considered relatively uniform [32,33].

To determine the airflow regime before the air entered the corn bed, the inner diameter of the measurement cylinder, *D_t_*, was selected as the characteristic length. The duct Reynolds number was calculated using the measured mean air velocity across the cross-section as follows:

(17) ReD=ρv¯Dtμ

where *Re_D_* is the duct Reynolds number; *ρ* is the air density, kg·m^−3^; $\bar{v}$ is the measured mean air velocity across the cross-section, m/s; *D_t_* is the inner diameter of the measurement cylinder, m; and *μ* is the dynamic viscosity of air, Pa·s.

At target inlet air velocities of 0.6, 0.8, and 1.0 m/s, the measured mean air velocities were 0.596, 0.802, and 1.032 m/s, respectively. The corresponding duct Reynolds numbers were approximately 5.95 × 10^3^, 8.00 × 10^3^, and 1.03 × 10^4^, respectively. All values were greater than 4000, indicating that the airflow was in the turbulent regime before entering the corn bed. The particle Reynolds number was further calculated using the equivalent particle diameter of corn, *D_p_* ≈ 7.65 mm, as the characteristic length:

(18) Rep=ρv¯Dpμ

The calculated particle Reynolds numbers at the three inlet air velocities were approximately 303, 408, and 525, respectively. These results indicate that inertial effects cannot be neglected when air flows through the corn bed. Therefore, the Ergun equation, which accounts for both viscous and inertial resistance, is appropriate for describing the ventilation resistance of the corn bed.

### 3.2. Analysis of Box–Behnken Experiment Results

The experimental results were processed and analyzed using Design-Expert 13, and the regression model comparing moisture content *A*, corn flow rate *B*, air speed *C*, corn pile height *D* and corn test layer porosity *Y* was obtained as follows:

(19) $$\begin{array}{l} Y=\;69.08899-0.512511A-125.30285B+12.70594C-0.142636D\\ +8.21632\mathrm{AB}+0.000799\mathrm{AC}-0.003500\mathrm{AD}-62.08750\mathrm{BC}+7.91100\mathrm{BD}\\ +0.093675\mathrm{CD}+0{.002974A}^{2}-1611{.40000B}^{2}-8{.09256C}^{2}-0{.007060D}^{2} \end{array}$$

To investigate the effects of the four factors and their interactions on corn-bed porosity, this study used the factor levels listed in Table 8 to establish a 29-run Box–Behnken design using Design-Expert 13. The measured corn-bed porosity under each experimental condition was used as the response variable *Y*. The complete experimental matrix and measured response values (*ε_r_*) are presented in Table 11, and the measured corn-bed porosity ranged from 57.8104% to 63.2989%.

Based on the 29 Box–Behnken experimental conditions listed in Table 10, the combined standard uncertainty of dynamic porosity was further calculated. It ranged from 0.546 to 7.662 percentage points, with an average value of 3.151 percentage points, while the relative combined standard uncertainty ranged from 0.876% to 13.062%, with an average value of 5.211%. The uncertainty components arising from pressure drop, airflow velocity, equivalent particle diameter, and corn-bed velocity ranged from 0.079 to 0.590, 0.502 to 0.823, 0.054 to 0.056, and 0.009 to 7.635 percentage points, respectively. Corn-bed velocity made the largest contribution in 24 of the 29 conditions. Because the local sensitivity of porosity to corn-bed velocity changed with moisture content, airflow velocity, and bed depth, the uncertainty component associated with this parameter differed markedly among the experimental conditions. The uncertainty of dynamic porosity therefore varied with the experimental conditions and was affected by multiple input quantities.

The ANOVA results for the quadratic regression model are presented in Table 12. The model was significant (*p* < 0.0001), with an *R*^2^ of 0.9600 and an adjusted *R*^2^ of 0.9201, indicating a good fit to the experimental data. The lack-of-fit *p*-value was 0.1101, indicating that the lack of fit was not significant. The ANOVA included all 29 experimental observations, giving 28 corrected total degrees of freedom. The five replicated center-point runs provided four degrees of freedom for pure error. The quadratic regression model accounted for 14 degrees of freedom, leaving 14 residual degrees of freedom, including 10 for lack of fit and 4 for pure error. Taken together, these statistical results show that the response surface model adequately describes the experimental data within the investigated ranges.

Further examination of the significance of each factor in Table 7 reveals that moisture content *A*, corn flow rate *B*, and pile height *D* all have a highly significant impact on the response value (*p* < 0.0001), while the linear influence of air speed *C* is not significant. In the interaction terms, *AB* and *BD* reach a significant level, indicating a significant interaction between moisture content *A* and corn flow rate *B*, and between corn flow rate B and pile height *D*. In the quadratic terms, *D*^2^ reaches a significant level, suggesting that pile height has a significant quadratic curvature effect on the response value. In terms of interaction effects, the influence of *AB* is greater than that of *BD*. Li et al. [25] also reported that moisture content, particle flow velocity, and bed thickness are important factors affecting the dynamic bed porosity of a flowing rice bed.

### 3.3. Effects of Multi-Factor Responses and Interactions on Corn-Bed Porosity

**Figure 14:**
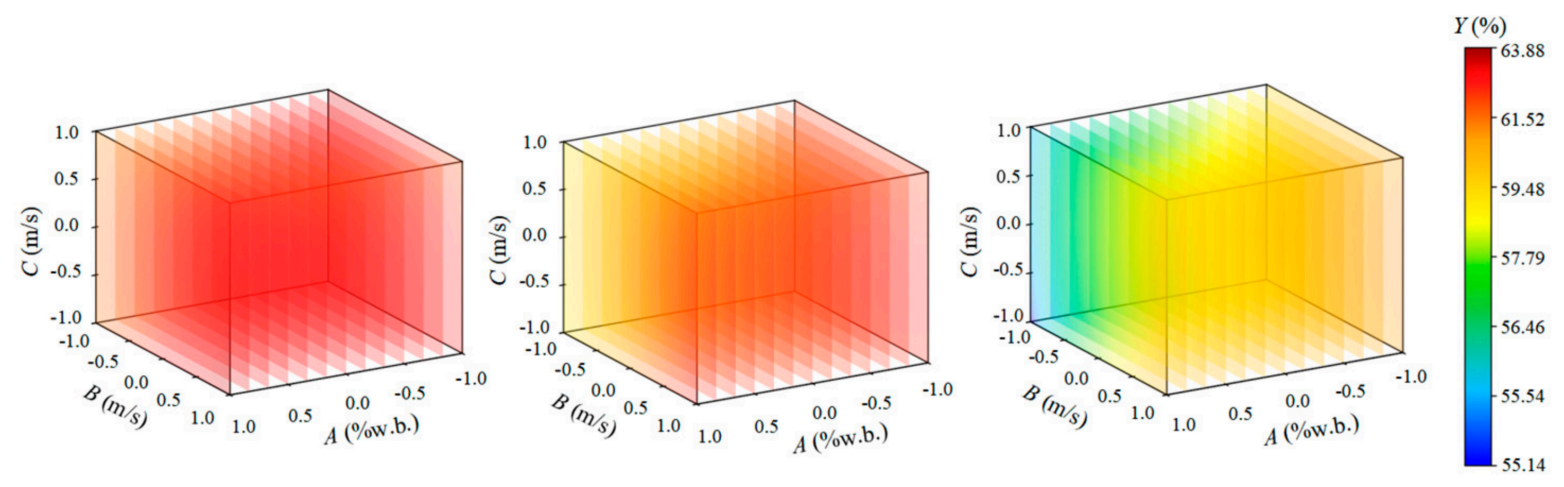
Four-dimensional response distributions of porosity to factors A, B, and C at different bed depths D.

Figure 14 shows the response distribution of *Y* to factors *A*, *B*, and *C* at different levels of bed depth *D*. The four-dimensional response distribution shows that, at a low corn velocity, increasing moisture content leads to a decrease in bed porosity, whereas, at a higher corn velocity, porosity increases due to enhanced particle rearrangement and collision frequency. Changes in corn moisture content will alter the adhesion, friction characteristics, and collision recovery behavior of the particle surface, and changes in corn flow rate will affect the rolling, sliding, collision, and rearrangement frequencies of the particles. As the moisture content decreases, the porosity of the corn bed layer gradually increases. The combined effect of these two factors causes the corn bed layer to continuously exhibit dynamic phenomena such as local compaction, local loosening, local bridging, re-crushing after agglomeration, surface undulation, and internal structure rearrangement during the experiment, and ultimately results in the fluctuation of the average porosity *Y*. To further determine that the particle flow state is an important factor affecting the porosity of the flow layer, Tong Jinjie et al. [34] mentioned that the porosity of the corn flow layer increases with the increase in the device opening. Boac et al. [35] established a DEM model for the mass flow of corn particles, which provided a methodological basis for further studying the effect of corn flow rate on porosity. He et al. [36], based on the discrete element method for the flow of corn-shaped particles, concluded that there is a correlation between the particle velocity distribution and the void fraction distribution.

**Figure 15:**
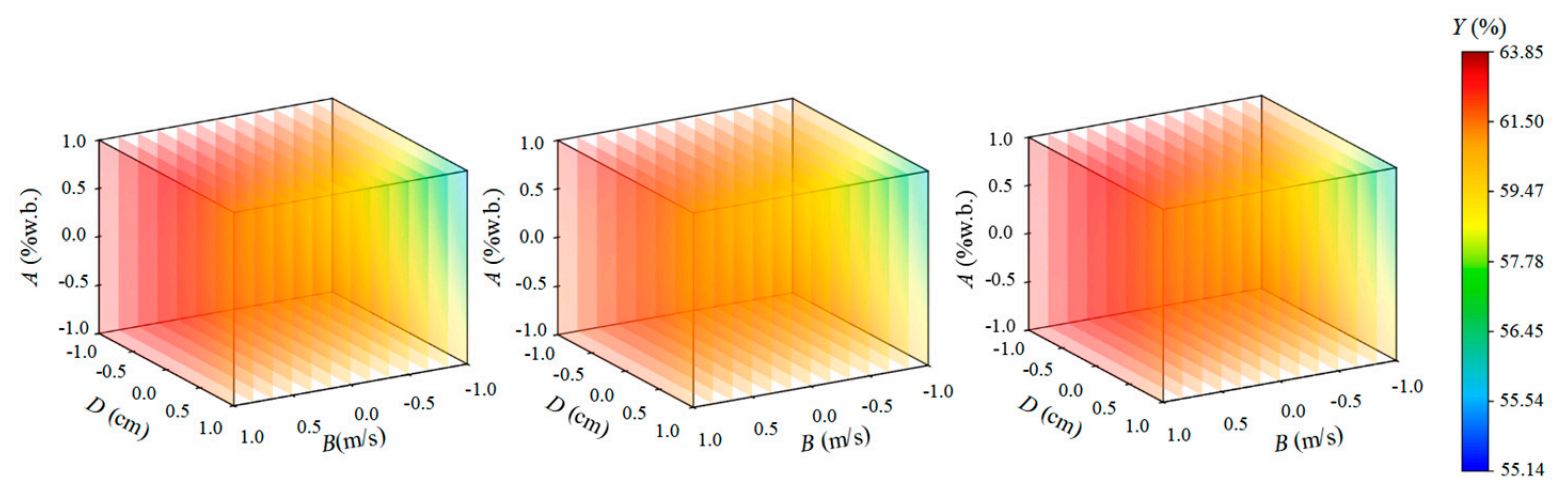
Four-dimensional response distributions of porosity to factors A, B, and D at different air velocities C.

Figure 15 shows the distribution of the response of *Y* to factors *A*, *B*, and *D* at different air velocities *C*. The four-dimensional response distributions indicate that the porosity *Y* of the dynamic corn layer decreases as the accumulation thickness *D* increases and increases as the corn flow rate B increases. Among them, the influence of *D* is stronger, and the effect of *B* mainly lies in promoting the rolling, collision and rearrangement of particles, partially weakening the compaction effect brought by the thick bed layer. Factors *B* and *D* exhibit an interaction effect on Y. The noticeable shift in the color distribution with increasing bed depth *D* suggests that the response of Y to the *B* and *D* interaction is mainly governed by the effect of *D*. In the experiment, the phenomenon is that, after the bed layer thickens, the pressure on the lower particles increases and the structure becomes more compact. When the flow rate increases, the particle movement becomes more intense and the local compact areas are dispersed, resulting in an increase in porosity.

In conclusion, three main factors affecting corn-bed porosity were identified: moisture content and bed depth mainly govern particle contact characteristics, while corn velocity controls the frequency of particle rearrangement. Their interactions determine the extent to which the dynamic corn bed shifts from a compacted to a loosened state, thereby influencing the variation in dynamic bed porosity. Therefore, dynamic bed porosity is not independently determined by a single factor, but rather results from the combined effects of particle contact characteristics, dynamic rearrangement, and bed compaction. This trend is generally consistent with previous studies on the porosity of flowing grain beds [25].

### 3.4. Comparison of Experimental and Model Results

Based on the Box–Behnken experimental scheme in Table 8, the EDEM–Fluent coupling simulation was repeated. Using Equation (5), the simulation value of the dynamic porosity of the corn bed (*ε_s_*) was obtained. The comparison of the experimental results is shown in Table 13. The relative error between the measured value (*ε_r_*) of the dynamic layer porosity of the corn and the simulation value (*ε_s_*), as well as the predicted value (*ε_p_*) of the mathematical model, is ±1% (in line with the instrument measurement error range, indicating that the measured porosity of the corn dynamic layer has certain reliability).

In practice, the experimental, simulated, and predicted values still exhibited certain discrepancies. These differences may be attributed to the variability in the size and shape of real particles, transient fluctuations within the dynamic bed, and simplifications of particle geometry and contact parameters in the numerical model. Previous studies have likewise shown that particle shape and the method used to characterize particle size can affect the structural characteristics of DEM particle assemblies [23,27]. Therefore, the model can reasonably capture the overall variation in bed porosity, although a certain degree of idealization remains.

### 3.5. Experimental Validation

Five independent validation experiments were conducted within the investigated factor ranges. For each validation condition, the corn-bed porosity was measured experimentally and compared with the value predicted by the response surface model. As shown in Table 14, the relative errors ranged from −0.60% to 0.69% and all absolute relative errors were below 1%. These results indicate that the predicted values were generally consistent with the measured values, suggesting that the established response surface model has good predictive accuracy.

### 3.6. Conduction of Local Simulation Analysis on the Corn Layer

**Figure 16:**
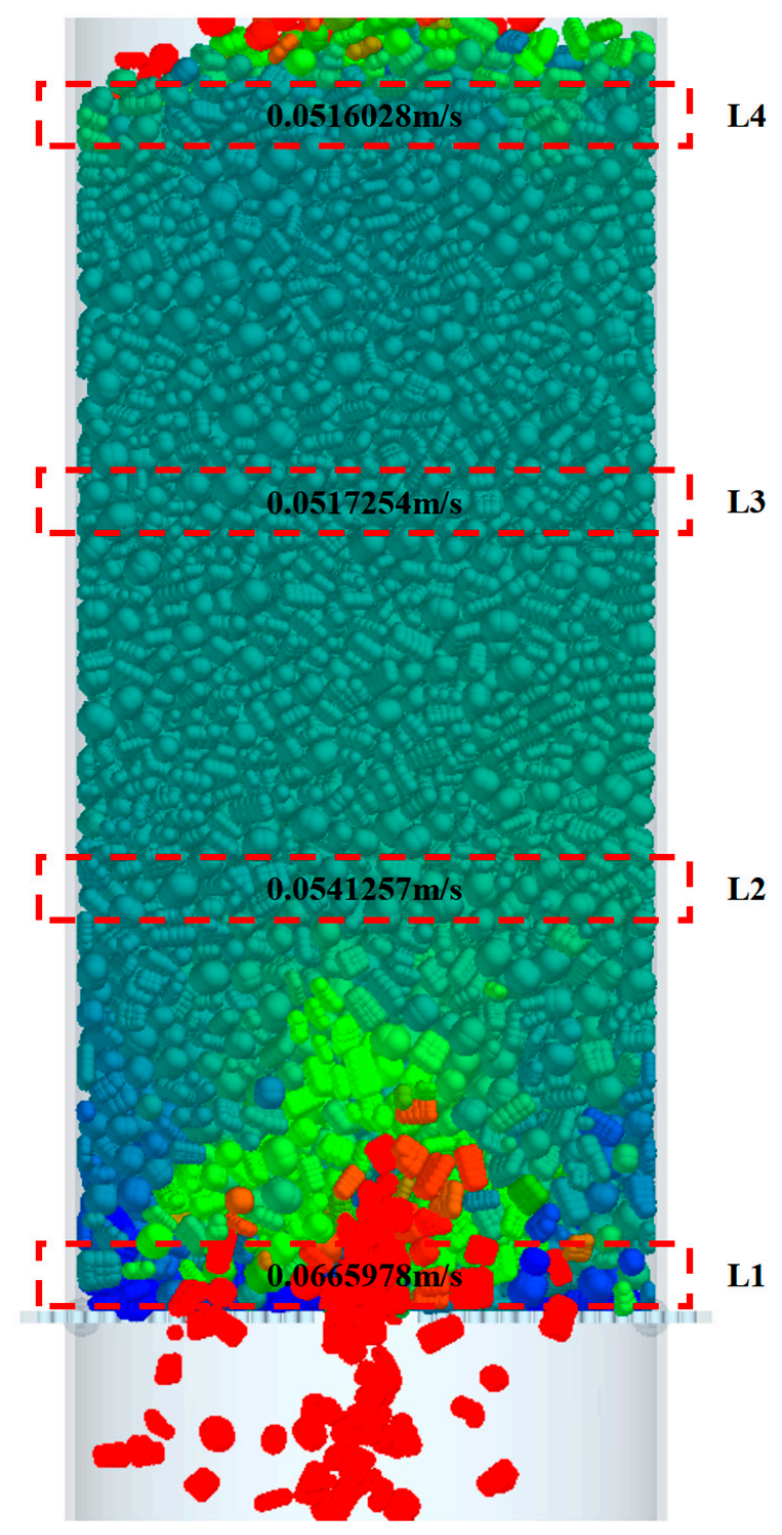
Four-layer local porosity measurement layer.

**Figure 17:**
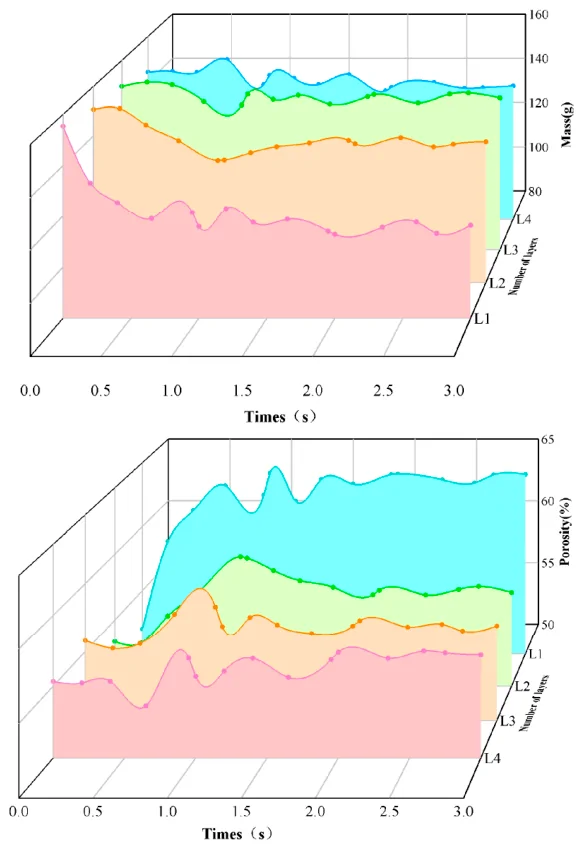
Local porosity test layer—corn quality and porosity.

In order to study the changes in the porosity of corn in actual drying equipment, during the simulation process, the measurement layer of bed porosity was divided into four local measurement layers, each with a thickness of 30 cm, as shown in Figure 16. In the EDEM software, the dynamic change quality data of the porosity measurement of each local layer was obtained, as shown in Figure 17. The dynamic change in the mass of corn was relatively stable in the L3 and L4 layers, with an average weight of approximately 141.247 g, and the corresponding porosity was lower. Conversely, the dynamic change in the mass of corn in the L1 layer was much greater than the other three layers and its porosity was also greater than the other three layers. This is because the flow rate of corn at the valve opening of the porosity measurement device in the L1 layer was faster compared to the other layers, causing the local porosity of the L1 layer to increase, resulting in a smaller number of particles and weight in this layer compared to the other three layers. The relatively high bed porosity in the L1 region is mainly attributed to the more intense particle motion and rearrangement near the outlet, whereas particle motion in the L2–L4 regions is comparatively stable, indicating pronounced spatial non-uniformity within the dynamic bed. Previous studies on maize grain beds have shown that pore structure and its spatial distribution can further influence airflow characteristics within the bed [2,20], leading to regional differences in air velocity as well as heat and moisture transfer.

### 3.7. Analysis of Correlation Coefficient Results

**Figure 18:**
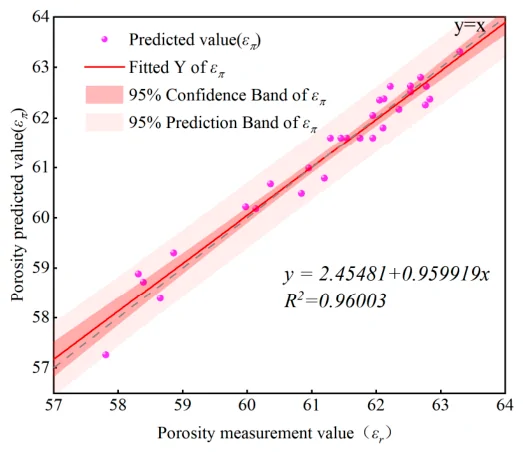
Correlation fitting curve graph.

Figure 18 shows that there is a strong consistency between the predicted porosity (*ε_p_*) and the measured porosity (*ε_r_*). The scatter points are generally distributed near the dashed line of *y* = *x*. The linear fitting result is *y* = 2.45481 + 0.959919*x*, with a determination coefficient of *R*^2^ = 0.96003. The model can well represent the variation pattern of the porosity in the dynamic layer of corn. From the closeness of the fitting line to the ideal isosurface, it can be seen that the deviation between the predicted results and the measured results is small, and no obvious systematic offset occurs. The 95% confidence band and the 95% prediction band are relatively narrow overall, and most sample points fall within the bands, further verifying that the regression model has good stability and high prediction accuracy [37]. The high correlation indicates that the model can effectively capture the overall variation trend in dynamic bed porosity. However, the current validation is primarily based on the macroscopically averaged bed porosity and therefore cannot fully characterize local pore connectivity, tortuosity, or near-wall structural features [1,38].

## 4. Conclusions

(1) This paper has established a dynamic layer porosity test platform for corn and, combined with the EDEM–Fluent coupling method, it has studied the changes in porosity under different moisture contents, air speeds, layer thicknesses and corn flow rates. Finally, it was concluded that the dynamic layer porosity of corn is not constant but shows obvious dynamic response characteristics with changes in working conditions. The obtained range of bed porosity is 57.8104–63.2989%.

(2) The analysis results obtained using the Design-Expert 13 software indicate that the moisture content (*M_c_*) and layer thickness (*D*) are the primary influencing factors for the variation in the dynamic porosity of corn. An increase in either of these two factors will cause the bed layer to tend towards a compacted state, resulting in a smaller porosity. An increase in the corn flow rate (*B*) is beneficial for particle rolling, collision, and rearrangement, thereby improving the looseness of the bed layer and increasing the porosity of the corn. The air speed (*C*) has a relatively weak impact on the overall porosity. The variance analysis results (*R*^2^ = 0.96003) indicate that the established mathematical model has a good fitting effect.

(3) The porosity trends obtained from the EDEM–Fluent coupled simulation were generally consistent with the experimental results, with a high fitting correlation. This indicates that the established model can reasonably describe the overall variation trend in dynamic bed porosity within the range of operating conditions investigated in this study.

(4) The pore volume variation pattern obtained from the local simulation of the EDEM–Fluent coupling indicates that the pore volume of the L1 layer is much larger than that of the other three layers. This is due to the maximum variation frequency of corn particles at the bottom discharge port. Conversely, the pore volume variations in the L2, L3, and L4 layers are relatively uniform.

(5) This study mainly focused on the relationship between particle motion and bed pore structure under specified moisture-content conditions. The continuous structural changes caused by particle moisture loss, shrinkage, and heat and mass transfer during the actual drying process were not considered. Future studies should further couple heat and mass transfer with dynamic changes in particle dimensions, and conduct experimental validation of local pore structures to improve the applicability of the model to practical grain drying processes.

## Figures and Tables

**Table 1:** The true density corresponding to different moisture content.

Moisture Content %w.b.	True Density g/cm^3^
13.03	1.4145
23.2	1.3520
33.37	1.2478

Note: The test samples at each moisture content level were composed of spherical-like, horse-tooth shape and rectangle corn particles in a 1:1:1 proportion.

**Table 2:** Air parameters.

Dry Bulb Temperature/°C	Relative Humidity/%	Air Density/kg·m^3^	Dynamic Viscosity of Air/Pa·s
18–22	48–52	1.204	1.81 × 10^−5^

**Table 3:** Valve opening and corn flow rate relationship table.

Moisture Content of Corn %w.b.	The Valve Opening mm	Velocity of Corn Flow Layer m/s
13.03%	19.80	0.03717 ± 0.00436
	23.19	0.05662 ± 0.01450
	24.04	0.06505 ± 0.00946
23.2%	21.10	0.04034 ± 0.00343
	22.63	0.05468 ± 0.00759
	24.32	0.06648 ± 0.00619
33.37%	20.51	0.04602 ± 0.00915
	23.19	0.05666 ± 0.00559
	25.17	0.06579 ± 0.00998

**Table 4:** Corn size measurement chart.

Particle Shape	Height h/mm	Lower Width b/mm	Lower Thickness e/mm	Upper Width a/mm	Upper Thickness d/mm
Spherical-like	9.580	9.212	8.281	8.280	7.746
Horse-tooth Shape	12.264	7.710	4.961	6.821	4.323
Rectangle	10.581	9.862	4.951	8.876	4.612

**Table 5:** Mesh quality assessment form.

Parameters	Values
Minimum unit quality	0.24
Maximum aspect ratio	9.081
Minimum orthogonal quality	0.205
Maximum skewness	0.795
Minimum tetrahedron folding	0.198
The number of mesh	2,136,376

**Table 6:** Material properties and contact parameters used in the numerical simulation [30].

Parameters	Values	Unit
Poisson ratio of corn kernels	0.25–0.35	-
Shear modulus of corn kernels	43.7–82.5	MPa
Elastic recovery coefficient between corn kernels	0.39–0.63	-
Coefficient of static friction between corn kernels	0.46–0.86	-
Coefficient of rolling friction between corn kernels	0.09–0.28	-
Poisson ratio of acrylic wall	0.35	-
Acrylic wall density	1.18	g/cm^3^
Shear modulus of acrylic wall	252	MPa
Elastic recovery coefficient of corn kernels and acrylic wall	0.38–0.67	-
Static friction coefficient between corn kernels and acrylic wall	0.37–0.62	-
Coefficient of rolling friction between corn kernels and acrylic wall	0.04–0.23	-

**Table 7:** Preliminary single-factor experimental scheme.

Number	*M_c_*% w.b.	*V_d_* (m/s)	*V_w_* (m/s)	*H* (cm)
1	13.03	0.05	0.8	30
2	23.2			
3	33.37			
4	23.2	0.04	0.8	30
5	23.2	0.05	0.8	30
6		0.06		
7	23.2	0.05	0.6	30
8			0.8	
9			1.0	
10	23.2	0.05	0.8	20
11				30
12				40

**Table 8:** Box–Behnken experimental scheme.

Code	*M_c_*%w.b.	*V_d_* (m/s)	*V_w_* (m/s)	*H* (cm)
1	13.03	0.04	0.6	20
0	23.2	0.05	0.8	30
−1	33.37	0.06	1.0	40

**Table 9:** Uncertainty of experiment parameters.

Parameter Name	Units	Uncertainty
Outlet air speed (*V_w_*)	m/s	±0.02
Air pressure difference (Δ*P*)	Pa	±1.50
Velocity of corn bed (*V_d_*)	m/s	±0.03
Valve opening (*l*)	mm	±0.50
Volume flow (*Q*)	m^3^/s	±0.002
Equivalent diameter of corn (*D_p_*)	mm	±0.03

**Table 10:** Air-velocity distribution and uniformity across measurement cross-section S at different target inlet air velocities.

Target Inlet Air Velocity, *V_w_*/(m · s^−1^)	Measured Air Velocity at Each Point on Cross-Section S/(m · s^−1^)					Measured Mean Air Velocity, $\bar{v}/(m\cdot s^{-1})$	Relative Deviation, *δ*/%	CV/(%)
	P1	P2	P3	P4	P5			
0.6	0.56	0.59	0.63	0.59	0.61	0.596	−0.67	4.38
0.8	0.75	0.78	0.86	0.8	0.82	0.802	0.25	5.17
1	0.92	1.01	1.12	1.04	1.07	1.032	3.20	7.23

**Table 11:** Box–Behnken experiment results.

Std	Run	*A* (%)	*B* (m/s)	*C* (m/s)	*D* (cm)	Δ*p*	*Y*
19	1	13.03	0.05	1	30	76.46	62.3565
29	2	23.2	0.05	0.8	30	102.45	61.9496
9	3	13.03	0.05	0.8	20	57.15	63.2989
28	4	23.2	0.05	0.8	30	111.66	61.2936
17	5	13.03	0.05	0.6	30	57.29	62.1225
26	6	23.2	0.05	0.8	30	88.69	61.7513
3	7	13.03	0.06	0.8	30	144.19	62.5361
16	8	23.2	0.06	1	30	115.81	62.1102
27	9	23.2	0.05	0.8	30	109.37	61.4542
23	10	23.2	0.04	0.8	40	99.9	57.8104
2	11	33.37	0.04	0.8	30	78.96	58.8643
4	12	33.37	0.06	0.8	30	144.11	62.691
1	13	13.03	0.04	0.8	30	74.93	62.0518
24	14	23.2	0.06	0.8	40	150.06	60.3655
10	15	33.37	0.05	0.8	20	57.9	62.5344
8	16	23.2	0.05	1	40	79.18	58.3152
15	17	23.2	0.04	1	30	59.9	59.9768
13	18	23.2	0.04	0.6	30	38.11	60.1348
25	19	23.2	0.05	0.8	30	102.45	61.5447
20	20	33.37	0.05	1	30	84.38	61.1989
6	21	23.2	0.05	1	20	54.41	61.9518
11	22	13.03	0.05	0.8	40	155.54	60.8437
12	23	33.37	0.05	0.8	40	154.74	58.6553
21	24	23.2	0.04	0.8	20	37.75	62.8327
5	25	23.2	0.05	0.6	20	45.58	62.7787
7	26	23.2	0.05	0.6	40	81.38	58.3927
14	27	23.2	0.06	0.6	30	117.08	62.7649
18	28	33.37	0.05	0.6	30	59.27	60.9584
22	29	23.2	0.06	0.8	20	82.62	62.2234

**Table 12:** Analysis of variance.

Source	Sum of Squares	df	Mean Square	F-Value	*p*-Value	
Model	64.63	14	4.62	24.02	<0.0001	significant
*A*	5.75	1	5.75	29.92	<0.0001	***
*B*	10.12	1	10.12	52.65	<0.0001	***
*C*	0.1287	1	0.1287	0.6694	0.427	
*D*	37.58	1	37.58	195.54	<0.0001	***
*AB*	2.79	1	2.79	14.53	0.0019	**
*AC*	0	1	0	0.0001	0.9942	
*AD*	0.5069	1	0.5069	2.64	0.1267	
*BC*	0.0617	1	0.0617	0.3209	0.58	
*BD*	2.5	1	2.5	13.02	0.0028	**
*CD*	0.1404	1	0.1404	0.7305	0.4071	
*A* ^2^	0.6137	1	0.6137	3.19	0.0956	
*B* ^2^	0.1684	1	0.1684	0.8763	0.3651	
*C* ^2^	0.6797	1	0.6797	3.54	0.081	
*D* ^2^	3.23	1	3.23	16.82	0.0011	**
Residual	2.69	14	0.1922			
Lack of Fit	2.43	10	0.2428	3.69	0.1101	not significant
Pure Error	0.2633	4	0.0658			
Cor Total	67.32	28				
Mean			61.23			
C.V. %			0.716			
R^2^			0.96			
Adjusted R^2^			0.9201			

** indicates highly significant; *** indicates extremely significant.

**Table 13:** Comparison of measured, simulated, and predicted corn-bed porosity.

Number	*ε_s_*/%	*ε_p_*/%	*ε_r_*/%	RE/%	
				Relative Error of *ε_s_* and *ε_r_*	Relative Error of *ε_p_* and *ε_r_*
1	62.5747	62.1701	62.3565	0.35	0.30
2	61.6894	61.5992	61.9496	−0.42	0.57
3	63.7926	63.3065	63.2989	0.78	−0.01
4	61.1281	61.5992	61.2936	−0.27	−0.50
5	62.5014	62.3805	62.1225	0.61	−0.42
6	61.8117	61.5992	61.7513	0.10	0.25
7	62.6862	62.5206	62.5361	0.24	0.02
8	61.6568	61.805	62.1102	−0.73	0.49
9	61.7492	61.5992	61.4542	0.48	−0.24
10	57.2843	57.2532	57.8104	−0.91	0.96
11	59.2587	59.2995	58.8643	0.67	−0.74
12	62.4653	62.8074	62.691	−0.36	−0.19
13	62.3745	62.3551	62.0518	0.52	−0.49
14	59.9792	60.6721	60.3655	−0.64	−0.51
15	62.7157	62.634	62.5344	0.29	−0.16
16	57.8018	58.8839	58.3152	−0.88	−0.98
17	60.0608	60.2166	59.9768	0.14	−0.40
18	59.8522	60.1754	60.1348	−0.47	−0.07
19	62.0555	61.5992	61.5447	0.83	−0.09
20	61.2092	60.789	61.1989	0.02	0.67
21	62.2987	62.0481	61.9518	0.56	−0.16
22	60.4239	60.4795	60.8437	−0.69	0.60
23	58.7843	58.3832	58.6553	0.22	0.46
24	62.4683	62.3743	62.8327	−0.58	0.73
25	63.2433	62.6299	62.7787	0.74	0.24
26	58.8051	58.7162	58.3927	0.71	−0.55
27	63.0097	62.2605	62.7649	0.39	0.80
28	60.3732	60.9928	60.9584	−0.96	−0.06
29	62.2545	62.6288	62.2234	0.05	−0.65

**Table 14:** Comparison of predicted and measured corn-bed porosity.

Number	*M_c_*/%	*V_d_*	*V_w_*	*H*	*ε_p_*/%	*ε_r_*/%	Relative Error %
1	26	0.04	0.7	21	61.9361	62.1513	0.35
2	27	0.05	0.9	22	62.2463	61.9726	−0.44
3	28	0.06	0.7	23	63.0473	63.4817	0.69
4	29	0.06	0.8	24	62.9929	62.615	−0.6
5	26	0.04	0.7	21	61.9361	62.0289	0.15

## Data Availability

The original contributions presented in this study are included in the article. Further inquiries can be directed to the corresponding author.
